# A Multilevel Person-Centered Examination of Teachers’ Workplace Demands and Resources: Links With Work-Related Well-Being

**DOI:** 10.3389/fpsyg.2020.00626

**Published:** 2020-04-08

**Authors:** Rebecca J. Collie, Lars-Erik Malmberg, Andrew J. Martin, Pamela Sammons, Alexandre J. S. Morin

**Affiliations:** ^1^School of Education, University of New South Wales, Sydney, NSW, Australia; ^2^Department of Education, University of Oxford, Oxford, United Kingdom; ^3^Substantive Methodological Synergy Research Laboratory, Department of Psychology, Concordia University, Montreal, QC, Canada

**Keywords:** job demands-resources theory, teacher well-being, latent profile analysis, multilevel, big data

## Abstract

Teachers’ healthy and effective functioning at work is impacted by the demands they face and the resources they can access. In this study, person-centered analysis was adopted to identify distinct teacher profiles of demands and resources. We investigated teachers’ experiences of two job demands (barriers to professional development and disruptive student behavior), two job resources (teacher collaboration and input in decision-making), and one personal resource (self-efficacy for teaching). Using data from the Teaching and Learning International Survey (TALIS) 2013, the study involved 6,411 teachers from 369 schools in Australia and 2,400 teachers from 154 schools in England. In phase one, latent profile analysis revealed five teacher profiles that were similar across the two countries: the Low-Demand-Flourisher (12%), Mixed-Demand-Flourisher (17%), Job-Resourced-Average (34%), Balanced-Average (15%), and Struggler (21%). The profiles were differently associated with two background characteristics (teacher gender and teaching experience) and two work-related well-being outcomes (job satisfaction and occupational commitment). In phase two, we extended our analysis to the school-level to identify school profiles based on the relative prevalence of the five teacher profiles within a school. Indeed, a yield of large scale datasets such as TALIS is that there are sufficient units at the school-level to enable institutional insights, beyond insights garnered at the individual teacher-level. Two school profiles that were similar in both countries were revealed: the Unsupportive school profile (58%) and the Supportive school profile (42%). The Supportive school profile was associated with higher school-average teacher job satisfaction and occupational commitment than the Unsupportive school profile. Taken together, the findings yield knowledge about salient teacher and school profiles, and provide guidance for possible interventions at the teacher- and school level.

## Introduction

Teaching work is complex. The extent to which teachers thrive at work involves a delicate balance between the demands placed upon them and the resources they can access to support them in their work (Hakanen et al., 2006). A growing body of research has examined the role of job demands (e.g., disruptive student behavior), job resources (e.g., social support), and personal resources (e.g., adaptability) in predicting teachers’ well-being at work (e.g., Desrumaux et al., 2015; Collie and Martin, 2017; Dicke et al., 2018; Skaalvik and Skaalvik, 2018). This prior work has tended to use variable-centered approaches (e.g., multiple/multivariate regression models within the structural equation modeling framework) that describe how the factors are interrelated (e.g., the association between job resources and well-being; Collie et al., 2018). Resting on the assumption of population-homogeneity, variable-centered approaches thus provide important information about relations at a sample-wide level and about the particular variables that could be targeted in broad intervention efforts. However, such research is less able to ascertain the extent to which there are different subpopulations of teachers identifiable based on commonly shared experiences of demands and resources reflecting population-heterogeneity, and whether there are particular combinations better aligned with well-being outcomes. To examine this, person-centered approaches, such as latent profile analyses, are ideally suited. Person-centered approaches identify distinct subpopulations (or profiles) of individuals who fare similarly on several factors. Person-centered approaches thus reveal knowledge of how intervention efforts can be tailored to the needs of each of these profiles.

A small, but growing body of research has conducted person-centered examinations of teachers’ experiences at work (e.g., Klusmann et al., 2008; Watt and Richardson, 2008; Simbula et al., 2012; Collie et al., 2015; Morin et al., 2015, 2017; Collie and Martin, 2017; Meyer et al., 2019; Perera et al., 2019). However, researchers have yet to consider job demands, job resources, and personal resources simultaneously. Moreover, the extent to which schools can be identified based on the prevalence of different demand-resource profiles among teachers remains largely unexamined. We suggest that these two gaps are important to address in order to ascertain the major categories of teachers who work in schools, the different combinations of demands and resources that characterize these subpopulations of teachers, and to inform policies and practice on how best to target intervention relevant to each distinct profile within and across schools. Such understanding is essential for promoting healthy and effective teachers and schools (e.g., Arens and Morin, 2016).

**FIGURE 1:**
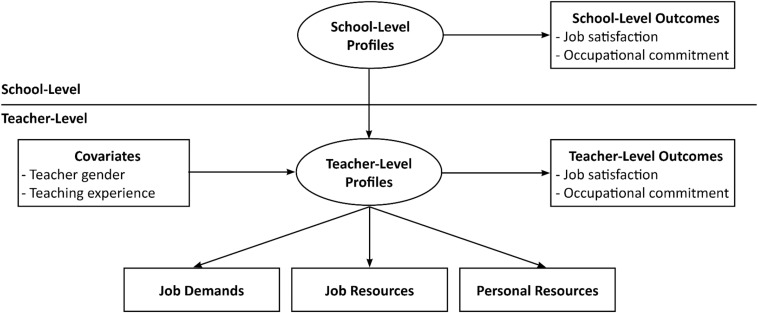
Hypothesized models tested in the study at the teacher- and school-level. In phase one of analysis, teacher-level profiles were identified based on the demands and resources. Then, tests of predictor associations with the profiles were conducted, followed by examination of mean differences between profiles on the teacher-level outcome. In phase two of analysis, school-level profiles were identified and then mean differences in the aggregated outcome were tested across school-level profiles. Not shown here are the tests of profile similarity that were conducted to compare the teacher-level results across Australia and England (see section “Materials and Methods).”

The aims of the current study, therefore, were to identify profiles of demands and resources experienced by teachers and then to explore the extent to which distinct profiles are predicted by teachers’ background characteristics and associated with meaningful differences in workplace well-being outcomes. We also investigated school-level profiles by identifying the proportion of the teacher-level profiles evident within different subpopulations of schools (i.e., school profiles), along with links to school-average well-being outcomes. Figure 1 demonstrates the models under examination. We harnessed job demands-resources theory (Bakker and Demerouti, 2017), and examined two job demands (barriers to professional development and disruptive student behavior), two job resources (teacher collaboration and input in decision-making), and one personal resource (self-efficacy for teaching). We examined these factors because together they reflect demands and resources that help or hinder teachers’ ability to undertake their work effectively (e.g., OECD, 2014; Skaalvik and Skaalvik, 2018). Of note, these factors have been shown to be implicated in teachers’ well-being in variable-centered analyses (e.g., Skaalvik and Skaalvik, 2018).

Data from the Teaching and Learning International Survey (TALIS) 2013 were used in our study. Key strengths of using large scale datasets like TALIS is that sampling is considered to be nationally representative, and sufficient numbers of schools are sampled to allow insights at the institutional level, beyond insights garnered at the individual teacher-level. Our study was conducted with teachers from Australia and England in order to provide evidence of the generalizability of these profiles, and to help guide national and international research, practice, and policy. Taken together, the findings have important implications for understanding demand-resource profiles among teachers and whether particular combinations are better aligned with well-being outcomes. The findings also have the potential to yield knowledge about salient school profiles and provide guidance for the development of appropriate intervention at the teacher- and school-level.

### Conceptual Framework

We rely on the job demands-resources (JD-R; Bakker and Demerouti, 2017) theory as our conceptual framework. This theoretical model highlights the idea that every job comprises varying types and levels of demands that impede employee functioning at work, as well as varying resources that support employee functioning at work (Bakker and Demerouti, 2017). Job demands (e.g., high workload) and job resources (e.g., strong social support) can be psychological, physical, social, or organizational in nature (Bakker and Demerouti, 2017). Although job resources are beneficial for employees’ motivation and well-being, job demands have the reverse association and are linked with a variety of undesirable outcomes, including burnout (Bakker and Demerouti, 2017). JD-R theory also stipulates the important role of personal resources, which are personal capacities that reflect employees’ potential to have influence on their working environment (e.g., self-efficacy; Bakker and Demerouti, 2017). Much like job resources, personal resources are beneficial for employees’ motivation and well-being at work (Bakker and Demerouti, 2017). In the current study, we examined job demands, job resources, and personal resources simultaneously.

### Demands and Resources Associated With Teachers’ Ability to Undertake Their Work

There are many job demands (e.g., time pressure), job resources (e.g., leadership support) and personal resources (e.g., adaptability) that impact teachers’ work (Collie et al., 2018; Skaalvik and Skaalvik, 2018). In the current study, we focused on several that are implicated in the extent to which teachers are able to effectively undertake their work (e.g., Collie et al., 2012), and that are also important for their well-being (e.g., Vangrieken et al., 2015).

The first job demand that we examined was *barriers to professional development*, which reflects teachers’ experience of factors preventing them from accessing the training necessary for their ongoing learning and development as a teacher. Barriers to professional development may include financial constraints, lack of appropriate opportunities, limited support from leadership, or limited time to complete such activities (Kwakman, 2003; OECD, 2009; Broadley, 2010). The second job demand was *disruptive student behavior*, which reflects behavior that makes it difficult for effective instruction to occur (e.g., students’ calling out or refusing to listen). Disruptive behavior has consistently been identified in research as challenging for teachers (e.g., Skaalvik and Skaalvik, 2018). Teachers who are unable to access professional development or who experience high levels of disruptive behavior in the classroom report reduced well-being (e.g., Collie et al., 2012; Skaalvik and Skaalvik, 2018). To our knowledge, researchers have yet to examine how these two job demands are interrelated. However, a positive association is assumed given that barriers to professional development may impinge on teachers’ ability to develop their capacities to effectively engage students and manage the classroom.

Turning attention to job resources, *teacher collaboration* involves the extent to which teachers work with their colleagues to plan, develop, teach, and/or assess student learning (OECD, 2014. Teacher collaboration helps teachers to undertake their work effectively because it can save time and introduce teachers to new ideas and resources (Reeves et al., 2017). *Teacher input in decision-making* refers to the extent to which the school provides teachers with opportunities to participate in and share responsibility for school-level decisions (OECD, 2014). Having input in decisions helps teachers to feel supported in their role (Collie et al., 2016) and it helps to ensure teachers’ needs are met (De Neve et al., 2015). Feeling supported and having needs met are important for helping teachers to undertake their work effectively (e.g., Taylor et al., 2008) and are also linked with teachers’ well-being at work (e.g., Vangrieken et al., 2015). Moreover, prior research suggests that these two job resources are moderately and positively correlated (e.g., Collie et al., 2012), which likely occurs because both collaboration and input reflect a school climate that is more collegial in relation to teaching and learning. Collegiality and positive relationships are known to be important for well-being (e.g., Ryan and Deci, 2017).

We also examined one personal resource—*self-efficacy for teaching*—which reflects teachers’ belief that they can bring about effective learning among students (Tschannen-Moran and Woolfolk Hoy, 2001). A large body of research has highlighted the important role of self-efficacy in many different countries (e.g., Fackler and Malmberg, 2016)—with both a focus on specific types of self-efficacy (for instructional practices, classroom management, and student engagement) and self-efficacy as a global construct (e.g., Collie et al., 2012; Granziera and Perera, 2019; Perera et al., 2019). The current study provided the opportunity to examine global levels of self-efficacy for teaching alongside other job demands and job resources to ascertain its role in teachers’ well-being.

Taken together, we thus focus on five factors that are salient in helping or hindering teachers to undertake their work effectively (e.g., Leithwood et al., 2008; Vangrieken et al., 2015). More precisely, these five factors encompass teachers’ interactions with students, other teachers, and school leaders, as well as teachers’ own professional growth and confidence. These different aspects reflect central components of teaching work (e.g., Collie et al., 2016) and are linked with teachers’ psychological functioning (e.g., Klassen et al., 2012). Teachers who have positive interactions with other school members, and who feel confident at what they do, are more likely to be satisfied with their work and committed to their profession (e.g., Collie et al., 2012). The opposite is true for teachers who have challenging interactions with students or who do not experience agency in relation to their professional growth (e.g., Skaalvik and Skaalvik, 2018). Notably, the bulk of prior research on these factors has been variable-centered in nature, revealing important knowledge about how these factors are associated with one another and important outcomes. However, variable-centered approaches are less able to speak to teacher profiles which might each reflect a different mix of demands and resources (i.e., high, medium, or low on different demands and resources)—a useful insight for targeted intervention and teacher support. This is where person-centered research is particularly informative.

### Identifying Teacher Profiles Through Person-Centered Research

Although variable-centered research harnessing JD-R theory is abundant (e.g., Simbula et al., 2012; Skaalvik and Skaalvik, 2018), person-centered research is only just emerging. We suggest that person-centered research has the potential to provide a theoretical contribution to JD-R theory because it allows simultaneous examination of the interplay between multiple factors. This interplay is relevant to a distinct process established in JD-R theory, the boosting process, which suggests that demands boost the impact of resources on workplace wellbeing (Bakker and Demerouti, 2017). More precisely, job resources play a more substantial role in promoting well-being when job demands are high. The boosting process is tested by examining interactions between demands and resources, and their joint association with well-being outcomes. Yet, it quickly becomes challenging, even impossible, to meaningfully interpret the results from interaction effects involving more than two or three interacting variables. Person-centered analysis provides another way of simultaneously considering the combined impact of multiple demands and resources, and allows a more nuanced overview of this combined role by revealing the specific interplay of factors that best characterize distinct segments (or profiles) of a population. For example, there may be a subpopulation of teachers for whom high levels of teacher collaboration and self-efficacy help to offset the detrimental role of disruptive student behaviors—resulting in levels of well-being that are similar or higher than in other groups who experience the same resources, but not the disruptive behavior (as per the boosting process in JD-R theory; Bakker and Demerouti, 2017).

Research is now investigating demands and resources using person-centered approaches, showing that various combinations of these factors exist among employees (e.g., Van den Broeck et al., 2012; Mäkikangas et al., 2018; Moeller et al., 2018). For example, Van den Broeck et al. (2012) examined several job demands (e.g., workload, emotional and cognitive demands) and job resources (e.g., social support, autonomy) among general employees. Their results revealed four demand-resource profiles: high demand-low resource, high demand/resource, low-demand/resource, and low demand-high resource. Employees in profiles with higher demands and lower resources reported greater burnout and lower engagement. To date, there appears to be very little research conducted among teachers. However, in one relevant teacher-focused study, Simbula et al. (2012) examined two job resources (professional development and collegial support) and two job demands (role ambiguity and over investment in work). The researchers identified three profiles: high demand/resource, high demand-low resource, and low resource-high demand. More generally, there is a growing number of teacher-focused studies that have involved examinations of well-being or motivation profiles, again revealing varying combinations that are associated with other important workplace outcomes (Klusmann et al., 2008; Watt and Richardson, 2008; Collie and Martin, 2017; Morin et al., 2017; Meyer et al., 2019).

In sum, there is emerging research undertaking person-centered examinations of demands and resources. However, to date, it appears that researchers have yet to simultaneously consider teachers’ perceptions of job demands, job resources, and personal resources. The aim of the current study was thus to extend the literature by considering these three facets simultaneously among teachers, and by focusing on factors that are central to teachers’ work and well-being. We suggest that this focus is important given that teaching work is uniquely distinct from other professional groups (e.g., different relationships with “clients”; Klassen et al., 2012) and given major concerns about teacher well-being worldwide (e.g., Skaalvik and Skaalvik, 2018).

### School-Level Phenomena and Their Association With Teachers’ Workplace Experiences

When factors play a significant role at the school-level, this indicates differences between schools that can then be a target for intervention. In research on teachers, the bulk of work has been conducted at the teacher-level. However, a growing body of work demonstrates the salience of considering factors at the school-level. For example, leadership style and school-average teacher collaboration have been associated with school-average teachers’ levels of organizational commitment (Duyar et al., 2013) and self-efficacy (Fackler and Malmberg, 2016). Although the variance explained by school-level factors can often be relatively modest, the school-level is nonetheless important to consider from a measurement perspective (Bliese et al., 2018) and given prior work demonstrating that school-level phenomena have a role to play in teachers’ outcomes (e.g., Duyar et al., 2013). Moving forward, it is important to extend this variable-centered knowledge to a person-centered understanding. Large secondary data sets like TALIS can play an important role in facilitating school-level research because they contain a sufficient number of schools to allow robust multilevel modeling. Multilevel person-centered approaches are able to ascertain the extent to which organizations can be identified based on the prevalence of different profiles among members (Mäkikangas et al., 2018). For example, Urick (2016) examined school-level profiles of leadership that were based on teacher-level profiles of leadership perceptions. Multilevel person-centered research, therefore, reveals the nature of different schools that can then be used to guide intervention efforts that target the specific needs of each school. For example, if there are schools that largely comprise teacher profiles reflecting low resources, then school-wide intervention may focus on increasing resources within those schools (in addition to interventions focused at the teacher-level).

### Do Teacher and School Characteristics Predict Profile Membership?

Background characteristics can provide additional understanding about the nature of profiles by revealing the extent to which profile membership is predicted by different teacher or school characteristics. Researchers have shown mixed findings on whether teacher gender predicts membership in demand-resource profiles (Simbula et al., 2012; Collie et al., 2015). Researchers have also shown that teaching experience is unrelated to membership in demand-resource profiles (Collie et al., 2015). This limited research provides preliminary understanding about the role of these background characteristics. However, more research is needed to see if these findings hold within additional samples and contexts. As such, we examined the role of teacher gender and teaching experience in predicting teacher-level profile membership.

### Are Profiles Linked With Teacher Work-Related Well-Being?

Teacher well-being is a multidimensional concept that reflects positive and healthy functioning in the workplace (Collie et al., 2016; Ryan and Deci, 2017). In the current study, we focused on two workplace outcomes that reflect experiences of work-related well-being: job satisfaction and occupational commitment. Job satisfaction involves employees’ feelings of contentment in relation to their work (Schleicher et al., 2011). Occupational commitment reflects employees’ attachment to their profession (Meyer et al., 1993). Variable-centered research has demonstrated the salience of a variety of job demands (e.g., time pressure), job resources (e.g., leadership support), and personal resources (e.g., adaptability) in predicting these two outcomes (e.g., Lee and Nie, 2014; Malinen and Savolainen, 2016; Skaalvik and Skaalvik, 2017; Collie et al., 2018). Emerging work is beginning to show that demand-resource profiles are differently linked with various workplace outcomes. For example, the Simbula et al. (2012) study introduced above identified three profiles of job demands and job resources, and demonstrated that these were linked with significantly different levels of workplace outcomes such as engagement and job satisfaction (the profiles with higher resources tended to display higher levels of the outcomes). In the current study, we extend prior research by examining novel job demands, job resources, and personal resources simultaneously—and in relation to both job satisfaction and occupational commitment. In addition, we also examined the extent to which the school-level profiles were associated with differences in school-average job satisfaction and occupational commitment.

### The Importance of Cross-National Research

There is a global recognition of the significance of demands and resources in impacting teachers’ well-being at work (e.g., Reeves et al., 2017; Dicke et al., 2018; Skaalvik and Skaalvik, 2018). An important and growing area of research, therefore, investigates teachers’ experiences across different countries (e.g., Watt et al., 2012; Fackler and Malmberg, 2016), and large scale datasets can provide important insights into this. Cross-national research can reveal similarities or differences in how teachers are faring across borders, and thus provide guidance for practice and policy at the national- and international-level (Watt et al., 2012). Despite growing awareness of the global relevance of teacher well-being, prior studies have typically been conducted within a single country (however, see Watt et al., 2012; Fackler and Malmberg, 2016).

The current research extends research in this area by examining the experiences of teachers in two English-speaking countries: Australia and England. There are some key similarities between the two countries. For example, Australia and England have similar schooling systems, ethnically diverse student populations, high inbound migration, and similar social stratification (e.g., Bulle, 2011; Scherer et al., 2016). In addition, major policy developments in the two countries over the past decade are relevant to teacher well-being. For example, both Australia and England have implemented national standardized testing for students, and increased scope of school evaluations (OECD, 2019). High-stakes tests and evaluations are known to be stressful for teachers (e.g., von der Embse et al., 2016). In addition, Australia and England have higher levels of disruptive behavior than the OECD average (OECD, 2019). Importantly, in both countries there is increasing unease about the demanding nature of teaching work and its impact on teachers’ well-being. Indeed, concerns about teacher attrition and burnout have been formally raised by governments in both countries over the past 12–18 months (e.g., UK Department for Education, 2018; Parliament of Australia, 2019). Similar policy-focused attention does not appear to be mirrored at a government-level in other English-speaking countries (e.g., Canada, the United States). Nonetheless, teacher well-being is an issue relevant to many other education contexts across the globe (e.g., United States, Hong Kong; Gallup, 2014; McInerney et al., 2018), and there are many commonalities in the demands and resources experienced by teachers worldwide (e.g., Fackler and Malmberg, 2016; Dicke et al., 2018; Skaalvik and Skaalvik, 2018). Thus, the results of the current study have the potential to provide knowledge for practice and research for teachers, schools, and educational systems internationally.

## Study Overview

In the current study, we identified demand-resource profiles among teachers and schools. We examined two job demands (barriers to professional development and disruptive behavior), two job resources (teacher collaboration and input in decision-making), and one personal resource (self-efficacy for teaching). In phase one of analysis, we identified demand-resource profiles among teachers in Australia and England. We then compared the solutions across the two countries to ascertain the generalizability of our results, as well as generalizability of links between the profiles and teachers’ background characteristics (gender, teaching experience) and two well-being outcomes (job satisfaction and occupational commitment). In phase two of analysis, we extended our examination to the school-level where we sought to identify school profiles based on the relative frequency of teacher profiles. We then compared these profiles across the two countries to verify the generalizability of these results, and tested whether the school profiles displayed different levels of school-average teacher job satisfaction and occupational commitment. Figure 1 illustrates the models under examination.

## Materials and Methods

### Sample and Procedure

The sample comprised 6,411 teachers from 369 schools in Australia and 2,400 teachers from 154 schools in England who participated in the Teaching and Learning International Survey (TALIS) 2013. TALIS is a survey run by the OECD every 5 years and was chosen for this study as it yields comprehensive and nationally representative data on demands and resources relevant to teachers’ work. Sample selection for TALIS 2013 involved a two-stage probability sampling design to ensure a representative sample of schools and of teachers within those schools (for full details see OECD, 2014).

Starting with the Australian sample, participating teachers were 57% female, had an average age of 43 (*SD* = 12) years, and had an average teaching experience of 16 (*SD* = 11) years. Most teachers (84%) were working full-time, and almost all (99%) had attained ISCED Level 5A (bachelor’s degree) or higher. The Australian teachers taught at ISCED Level 2 (lower secondary) and/or ISCED Level 3 (upper secondary). Just over half (55%) of the participating schools were publicly managed, and most (74%) had fewer than one-third students from low-SES backgrounds. Most schools (62%) had a male principal with an average age of 55 (*SD* = 7) years and an average experience as a principal of 9 (*SD* = 7) years. The schools were located in hamlets/villages (5%; <3,000 people), small towns (7%; 3,000–15,000 people), towns (16%; 15,000–100,000 people), cities (26%; 100,000–1 million people), and large cities (46%; >1 million people). There were on average 18 (*SD* = 5) teachers per school.

Participating teachers from England were 63% female, had an average age of 39 (*SD* = 10) years, and had an average teaching experiences of 12 (*SD* = 9) years. Most teachers (87%) were working full-time, and almost all (97%) had attained ISCED Level 5A (bachelor’s degree) or higher. The entire English sample taught at ISCED Level 2 (lower secondary). Just over half (55%) of the participating schools were publicly managed, and most (76%) had fewer than one-third of their students from low-SES backgrounds. Most schools (64%) had a male principal with an average age of 50 (*SD* = 7) years and an average experience as a principal of 7 (*SD* = 6) years. The schools were located in hamlets/villages (4%), small towns (18%), towns (41%), cities (20%), and large cities (16%). There were on average 16 (*SD* = 4) teachers per school.

### Measures

Measures were drawn from the 2013 TALIS Teacher Questionnaire (OECD, 2014; see Supplementary Material for items). All variables were modeled at the teacher-level. The teacher well-being outcomes (job satisfaction and occupational commitment) were also modeled at the school-level.

#### Job Demands

*Barriers to professional development* was assessed with items from the TALIS “Barriers to Professional Development” scale (6 items; e.g., “Professional development is too expensive/unaffordable”). Items were scored on a scale from 1 (Strongly disagree) to 4 (Strongly agree). Reliability was assessed with coefficient omega^1^ and was adequate for the Australian (ω = 0.74) and English (ω = 0.74) samples. The scale displayed 7% variance (intraclass correlation [ICC] = 0.07) at the school-level. Although this is somewhat modest, it does warrant multilevel analyses (Bliese et al., 2018).

*Disruptive student behavior* was assessed with items from the TALIS “Your Teaching” scale (3 items; “When the lesson begins, I have to wait quite a long time for students to quiet down,” “I lose quite a lot of time because of students interrupting the lesson,” and “There is much disruptive noise in this classroom”). Items were scored on a scale from 1 (Strongly disagree) to 4 (Strongly agree). Reliability was satisfactory (ω_Aus_ = 0.89; ω_Eng_ = 0.90) and the scale demonstrated adequate variance at the school-level (ICC = 0.14).

#### Job Resources

*Teacher collaboration* was assessed with items from the TALIS “Teaching in General” scale (3 items; “On average, how often do you do the following in this school? ‘Exchange teaching materials with colleagues,’ ‘Engage in discussions about the learning development of specific students,’ and ‘Work with other teachers in my school to ensure common standards in evaluations for assessing student progress”’). Items were scored from 1 (Never) to 6 (Once a week or more). Reliability estimates were adequate (ω_Aus_ = 0.75; ω_Eng_ = 0.73) and the scale demonstrated modest, but adequate, variance at the school-level (ICC = 0.05; Bliese et al., 2018).

*Teacher input in decision-making* was assessed with items from the TALIS “School Climate” scale (3 items; “This school provides staff with opportunities to actively participate in school decisions,” “This school has a culture of shared responsibility for school issues,” and “There is a collaborative school culture which is characterized by mutual support”). Items were scored on a scale from 1 (Strongly disagree) to 4 (Strongly agree). Reliability was satisfactory (ω_Aus_ = 0.86; ω_Eng_ = 0.87) and the scale demonstrated adequate variance at the school-level (ICC = 0.14).

#### Personal Resources

*Teacher self-efficacy* was assessed with items from the TALIS “Teaching in General” scale that encompasses three types of self-efficacy: self-efficacy for classroom management (4 items; “In your teaching, to what extent can you do the following? ‘Control disruptive behavior in the classroom’), self-efficacy for instruction (4 items; “In your teaching, to what extent can you do the following? ‘Craft good questions for my students’), and self-efficacy for student engagement (4 items; “In your teaching, to what extent can you do the following? ‘Help my students value learning’). Items were scored on a scale from 1 (Not at all) to 4 (A lot). Reliability was satisfactory (ω_Aus_ = 0.86; ω_Eng_ = 0.86) and there was modest, but adequate variance at the school-level (ICC = 0.05; Bliese et al., 2018). Preliminary analyses indicated that the three different self-efficacy factors co-occurred at similar levels across profiles. For reasons of parsimony and because the self-efficacy factors were quite strongly intercorrelated (*r*’s = 0.64–0.72), self-efficacy was modeled as a higher-order factor.

#### Outcomes

*Job satisfaction* and *occupational commitment* were assessed with items from the TALIS “About Your Job” scale. For job satisfaction, 3 items were used (“I enjoy working at this school,” “I would recommend my school as a good place to work,” and “All in all, I am satisfied with my job”). For occupational commitment, 4 items were used (“The advantages of being a teacher clearly outweigh the disadvantages,” “If I could decide again, I would still choose to work as a teacher,” “I regret that I decided to become a teacher” [reverse coded], “I wonder whether it would have been better to choose another profession” [reverse coded]). Items for both scales were scored on a scale from 1 (Strongly disagree) to 4 (Strongly agree). Because the outcomes were modeled at both the teacher- and school-level, we assessed reliability at both levels. Reliability was satisfactory at the teacher (ω_Aus_ = 0.83 and ω_Eng_ = 0.86 for job satisfaction; ω_Aus_ = 0.85 and ω_Eng_ = 0.87 for occupational commitment) and school (ω_Aus_ = 0.96 and ω_Eng_ = 0.97 for job satisfaction; ω_Aus_ = 0.99 and ω_Eng_ = 0.98 for occupational commitment) levels. The two scales also demonstrated adequate variance at the school-level (ICC = 0.14 for job satisfaction; ICC = 0.04 for occupational commitment; Bliese et al., 2018).

#### Teacher Characteristics

*Teacher gender* was coded 0 for female, 1 for male. *Teaching experience* was a continuous variable measured in years.

## Data Analysis

All analyses were conducted using M*plus* 8.4 (Muthén and Muthén, 2017). In our analyses, teacher (TCHWGT) and school weights (SCHWGT) were applied to adjust teacher and school scores to account for the probabilities of selection and participation at the different stages of sampling (see OECD, 2014 for full details about the weighting procedure). In addition, the clustering of teachers within schools was accounted for in single-level modeling by using the cluster command in M*plus*. The robust maximum likelihood (MLR) estimator was used in all models. Missing data were 5–8% for all variables (except disruptive student behavior, which was 17%). Missing data were handled with full information maximum likelihood procedures.

### Preliminary Analyses

Confirmatory factor analyses (CFA) were run for each country separately to obtain estimates of correlations among the two background characteristics, the five demands and resources, and the two outcomes (see the Supplementary Material for further details). We also ran measurement invariance tests using multigroup CFA to ensure that the ratings obtained in the Australian and English samples could be considered to be comparable. These models involved the latent factors for the five demands and resources (self-efficacy was modeled as a higher-order factor defined from three first-order factors), which were directly estimated from their items. We examined four models that were progressively more restrictive: configural (allowing all parameters to be freely estimated across the two countries), metric (loadings fixed to equality across countries), scalar (loadings and intercepts fixed across countries), and latent variance-covariance (loadings, intercepts, variances, and covariances fixed across countries) invariance models. We looked for changes in RMSEA across the models of 0.015 or less and for changes in CFI and TLI of 0.01 or less to establish invariance (Cheung and Rensvold, 2002; Chen, 2007). Factor scores were saved from the most constrained measurement model that was found to be invariant (with background characteristics and mean scores of the outcomes included as auxiliary variables). These factors scores were used in the latent profile analyses (LPA). More precisely, we used factor scores for the two job demands (barriers to professional development and disruptive behavior), the two job resources (teacher collaboration and input in decision-making), and the personal resource (self-efficacy for teaching) as profile indicator variables in the single-level and multilevel LPAs described below. Using these factor scores, we then ran a multigroup (across countries) measurement model in order to standardize the L1 and L2 sampling weights separately for each country. This step was necessary because of the way the weights were prepared in the original data (i.e., separately by country; syntax for this step is available in the Supplementary Material). The within-country standardized weights were saved (“savedata”) and used in all analyses as outlined below.

#### Single-Level LPA

For the single-level LPA conducted at the teacher level, we tested a range of solutions involving 1 through 8 profiles separately for Australia and England. Profile indicator variables were standardized (*M* = 0, *SD* = 1) for each country. All analyses relied on an assumption of conditional independence, meaning that any covariance between indicators is assumed to be entirely explained by the latent profile variable, given that we did not have any *a priori* theoretical or empirical reason for relaxing this assumption (e.g., Meyer and Morin, 2016). Means and variances were allowed to differ across indicator variables and profiles.

Each model was estimated using at least 6,000 random start values, each allowed 100 iterations, and 100 final stage optimizations. We also verified that the best log-likelihood value was properly replicated for all models. Several indices were used to assess the fit of the different models. For the Akaike Information Criteria (AIC), Consistent Akaike Information Criteria (CAIC), Bayesian Information Criteria (BIC), and sample-size-adjusted Bayesian Information Criteria (SSA-BIC) smaller values reflect better fit. The *p*-value of the adjusted Lo–Mendell–Rubin Likelihood Ratio Test (*p*LMR) allows comparison of a *k*-profile model with a *k*−1 profile model to see if the former model results in an improvement in fit relative to the latter. Finally, we created elbow plots of the AIC, CAIC, BIC, and SSA-BIC indices. In these plots, the profile at which point the slope noticeably flattens is another indicator of an appropriate solution (Morin et al., 2016). We also report entropy where values closer to 1 reflect greater profile separation. Alongside fit indices, we used parsimony, conceptual relevance, and statistical adequacy to help determine the optimal solution for each country.

After determining the optimal solution for each country, we next undertook tests of profile similarity to determine the extent to which the profile solutions could be considered to be comparable across Australia and England (Morin et al., 2016). These tests were conducted in the following sequence (Morin et al., 2016): configural (testing that the appropriate number of profiles was the same in the two countries), structural (constraining the means of the profile indicators to be the same across the two countries), dispersion (constraining the variance of the profile indicators to be the same across the two countries), and distributional (constraining the relative size of the profiles to be equal across countries). As recommended by Morin et al. (2016) we considered that profile similarity was supported when two indicators out of the CAIC, BIC, and SSA-BIC were lower (or equal) for the more constrained models relative to the previous model in the sequence. More precisely, tests of profile similarity seek to assess whether observed variations in person-centered results represent meaningful cross-country differences or whether they can simply be assumed to reflect random sampling variations. In other words, person-centered interpretations should be based on examination of the most similar solution rather than on a less accurate check of solutions separately estimated across countries (Morin et al., 2016).

Following these initial profile similarity tests, two additional tests of similarity were also conducted in order to examine the equivalence of the associations between the predictors (i.e., background characteristics) and likelihood of profile membership (predictive similarity), as well as the equivalence of the associations between profile membership and the outcomes (explanatory similarity) across countries (Morin et al., 2016). For these tests, predictors and outcomes were added to the most similar model from the previous sequence. We first ran an unconstrained model in which associations between the profiles, and the predictors or outcomes were allowed to vary across country. Then, a second model constrained these associations to be equal across country. The precise role of predictors (i.e., gender, teaching experience) was further examined using a multinomial logistic regression, using one latent profile as a reference group (Vermunt, 2010). Unstandardized beta coefficients, standard errors, and odds ratios (ORs) are presented from this analysis. ORs with a value greater than one indicate the increased likelihood of membership in a profile (compared with a reference profile) for every unit of increase in the predictor variable. The reverse is true for ORs < 1. Outcome levels were compared across profiles using the M*plus* MODEL CONSTRAINT option, which relies on the multivariate delta method for tests of statistical significance (e.g., Muthén and Muthén, 2017).

#### Multilevel LPA

In phase two, our aim was to extend the teacher-level (level 1, L1) findings to consider the extent to which school-level (level 2; L2) profiles could be identified. More precisely, these analyses sought to identify school profiles characterized by distinct proportions of teacher profiles. Thus, rather than estimating profiles (as in our single level analyses) based on the means and variance of profile indicators, this second set of analyses identified school profiles based on the relative frequency of the various categories of the L1 latent profiles (thus mathematically corresponding to a L2 latent class analysis; e.g., Morin and Litalien, 2017). To maintain the stability, and cross-country equivalence, of the previously identified teacher-level profiles, we relied on the manual 3-step approach described by Litalien et al. (2019; also see Morin and Litalien, 2017). This approach was necessary given the way the L2 analyses were conducted, allowing the L1 profiles to be “predicted” by the L2 profiles, thus making it impossible to implement any direct constraint on the relative frequency of occurrence of the L1 profiles across countries (i.e., distributional similarity; see Morin and Litalien, 2017). Additional details on the implementation of this approach are provided in the Supplementary Material.

Multilevel LPA solutions including 1 to 8 school-level profiles were first estimated separately in both countries. Each model was estimated using at least 6,000 random start values, each allowed 100 iterations, and 100 final stage optimizations. We also verified that the best log-likelihood value was properly replicated for all models. Model selection relied on the same criteria as used for the single level LPA, with the exception of the LMR, which is not available for multilevel LPA. After determining the optimal solution for each country, we ran L2 profile similarity tests to determine the extent to which the L2 profiles could be considered to be comparable across Australia and England. For this, we extrapolated upon the tests developed by Morin et al. (2016) for single level LPA as well as those developed by Eid et al. (2003) for single level latent class analyses. These tests were conducted in the following sequence: configural (testing that the appropriate number of L2 profiles was the same in the two countries), structural (constraining the relative frequency of the L1 profiles defining the L2 profiles to be the same across the two countries), and distributional (constraining the relative size of the L2 profiles to be equal across countries). Finally, we tested for L2-explanatory similarity by adding school-average outcomes to the most similar model determined in the L2 profile similarity tests. Annotated M*plus* input files for the estimation of these models are provided in the Supplementary Material.

## Results

### Preliminary Analyses

Table 1 displays reliability coefficients and descriptive statistics for each sample at L1 and L2. These data indicate appropriate reliability. Within-country CFAs provided correlations between all variables examined in the study (the resulting correlation matrix is available in the Supplementary Material). Tests of measurement invariance supported the equivalence of the factor loadings, item intercepts, latent variances, and latent covariances across countries with all ΔRMSEA ≤ 0.015 (Chen, 2007), and ΔCFI and ΔTLI ≤ 0.01 (Cheung and Rensvold, 2002; i.e., configural invariance RMSEA = 0.04, CFI = 0.93, and TLI = 0.92; metric invariance RMSEA = 0.04, CFI = 0.93, and TLI = 0.93; scalar invariance RMSEA = 0.04, CFI = 0.93, and TLI = 0.92; latent variance-covariance invariance RMSEA = 0.04, CFI = 0.93, and TLI = 0.92). Factor scores were thus obtained from the most constrained model (latent variance-covariance invariance) to use in the LPA and sampling weights were standardized within each country.

**TABLE 1:** Reliabilities and descriptive statistics for Australia and England.

	Australia			England		
	ω_Aus_	*M*	*SD*	ω_Eng_	*M*	*SD*
**Teacher-level**						
Gender	—	1.42	0.49	—	1.36	0.48
Teacher experience	—	16.02	11.06	—	12.71	9.42
Barriers to professional development	0.74	2.24	0.58	0.74	2.23	0.58
Disruptive student behavior	0.89	1.98	0.74	0.90	1.95	0.75
Teacher collaboration	0.75	4.91	1.04	0.73	4.72	1.06
Teacher input	0.86	2.66	0.67	0.87	2.64	0.69
Teacher self-efficacy	0.86	3.27	0.48	0.86	3.37	0.45
Job satisfaction	0.83	3.23	0.58	0.86	3.06	0.63
Occ. commitment	0.85	3.18	0.63	0.87	3.13	0.66
**School-level**						
School-average job satisfaction	0.96	3.22	0.26	0.97	3.04	0.26
School-average occ. commitment	0.99	3.18	0.23	0.98	3.12	0.20

*ω = Coefficient omega. Occ. commitment = Occupational commitment.*

### Single-Level LPA

The fit statistics associated with the solutions including 1 to 8 profiles estimated separately in Australia and England are reported in Table 2. For both countries, the AIC, CAIC, BIC, SSA-BIC decreased as additional profiles were added. For Australia, the *p*LMR supported the 6-profile solution. For England, the *p*LMR failed to support any specific solution. Elbow plots were also consulted for both countries (see the Supplementary Material) and showed a slight flattening of the slope around 5-profiles in both countries. Thus, the fit statistics themselves failed to pinpoint any specific solution in both countries, but suggest that the optimal solution might be close to five profiles in both countries. Thus, to support the selection of the optimal solution, we considered the conceptual relevance, parsimony, and meaningfulness of the 5-profile solution, together with that of the adjacent 4- and 6-profile solutions. A first noteworthy observation was that examination of these solutions already revealed a high level of similarity across country, thus providing early support for configural similarity. When we compared the 4-profile solution with the 5-profile solution, this examination revealed that the additional profile was meaningful in its own right in both countries, presenting a well-differentiated shape relative to the other profiles. However, adding a sixth profile did not appear to contribute additional information to the solution, simply resulting in the arbitrary division of one of the profiles into to smaller profiles presenting a similar shape. The 5-profile solution was thus retained for both countries, and submitted to more systematic tests of profile similarity.

**TABLE 2:** Fit statistics and entropy for Australia and England.

	Log-likelihood	Free parameters	AIC	CAIC	BIC	SSA-BIC	Entropy	*p*LMR
**Australia – Single-level**								
(1) Profile	−45484.08	10	90988.15	91065.81	91055.81	91024.03	—	—
(2) profiles	−42500.10	21	85042.20	85205.28	85184.28	85117.55	0.84	<0.001
(3) Profiles	−40642.59	32	81349.19	81597.69	81565.69	81464.00	0.88	<0.001
(4) Profiles	−39425.46	43	78936.92	79270.85	79227.85	79091.21	0.79	<0.001
(5) Profiles	−38387.83	54	76883.66	77303.01	77249.01	77077.41	0.80	<0.001
(6) Profiles	−37853.14	65	75836.28	76341.06	76276.06	76069.50	0.81	<0.001
(7) Profiles	−37347.07	76	74846.15	75436.35	75360.35	75118.84	0.80	*ns*
(8) Profiles	−36786.70	87	73747.40	74423.02	74336.02	74059.56	0.81	<0.001
**Australia – Multilevel**								
(1) Profile	−9778.15	4	19564.30	19595.36	19591.36	19578.65	0.68	—
(2) Profiles	−9703.77	9	19425.54	19495.43	19486.43	19457.83	0.64	—
(3) Profiles	−9684.58	14	19397.16	19505.88	19491.88	19447.39	0.61	—
(4) Profiles	−9672.83	19	19383.66	19531.21	19512.21	19451.83	0.61	—
(5) Profiles	−9665.04	24	19378.07	19564.45	19540.45	19464.19	0.63	—
(6) Profiles	−9660.65	29	19379.31	19604.51	19575.51	19483.36	0.62	—
(7) Profiles	−9657.87	34	19383.75	19647.79	19613.79	19505.74	0.61	—
(8) Profiles	−9655.10	39	19388.20	19691.07	19652.07	19528.14	0.60	—
**England – Single-level**								
(1) Profile	−17027.26	10	34074.53	34142.36	34132.36	34100.59	—	—
(2) Profiles	−15929.48	21	31900.96	32043.40	32022.40	31955.68	0.87	<0.001
(3) Profiles	−15191.69	32	30447.38	30664.44	30632.44	30530.77	0.90	<0.001
(4) Profiles	−14717.76	43	29521.52	29813.20	29770.20	29633.57	0.80	0.01
(5) Profiles	−14321.04	54	28750.09	29116.38	29062.38	28890.81	0.81	<0.001
(6) Profiles	−14059.53	65	28249.05	28689.96	28624.96	28418.44	0.83	<0.001
(7) Profiles	−13889.55	76	27931.09	28446.62	28370.62	28129.15	0.83	0.003
(8) Profiles	−13730.97	87	27635.95	28226.09	28139.09	27862.67	0.84	0.003
**England – Multilevel**								
(1) Profile	−3746.68	4	7501.37	7528.50	7524.50	7511.79	0.67	—
(2) Profiles	−3711.05	9	7440.09	7501.14	7492.14	7463.55	0.69	—
(3) Profiles	−3703.07	14	7434.14	7529.10	7515.10	7470.62	0.70	—
(4) Profiles	−3697.53	19	7433.06	7561.94	7542.94	7482.57	0.65	—
(5) Profiles	−3695.35	24	7438.69	7601.49	7577.49	7501.24	0.67	—
(6) Profiles	−3694.20	29	7446.40	7643.12	7614.12	7521.98	0.69	—
(7) Profiles	−3693.21	34	7454.42	7685.05	7651.05	7543.02	0.69	—
(8) Profiles	−3692.64	39	7463.27	7727.82	7688.82	7564.91	0.70	—

*AIC = Akaike Information Criteria. CAIC = Consistent Akaike Information Criteria. BIC = Bayesian Information Criteria. SSA-BIC = sample-size-adjusted Bayesian Information Criteria. pLMR = Lo–Mendell–Rubin Likelihood Ratio Test. ns = non-significant.*

**FIGURE 2:**
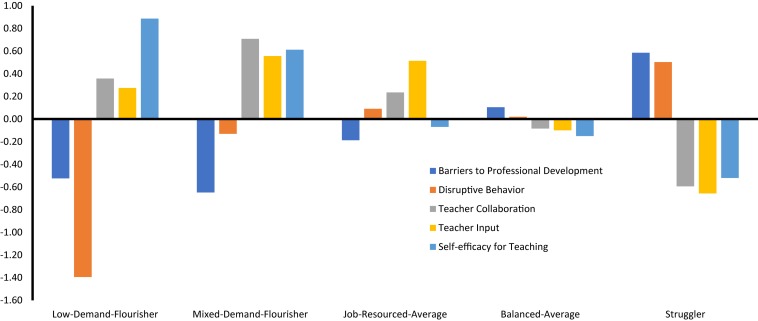
Single-level LPA results of distributional similarity showing teacher profiles for both countries.

The results from the tests of profile similarity conducted across countries are reported in Table 3. These results revealed that, each step of the sequence of similarity tests resulted in a decrease in the value of the CAIC, BIC, and SSA-BIC, thus supporting the complete (configural, structural, dispersion, and distributional) similarity of the solution across countries. A graphical representation of this final 5-profile solution of distributional similarity is presented in Figure 2, and detailed results are reported in the Supplementary Material.

**TABLE 3:** Tests of profile similarity across Australia and England.

	Log-Likelihood	Free parameters	AIC	CAIC	BIC	SSA-BIC	Entropy
**Single-level LPA**							
Configural	−58082.70	109	116383.39	117264.52	117155.52	116809.14	0.87
Structural (means)	−57975.96	84	116119.92	116798.96	116714.96	116448.02	0.84
Dispersion (means and variances)	−58004.77	59	116127.55	116604.49	116545.49	116358.00	0.86
Distributional (means, variances, probabilities)	−58009.74	55	116129.49	116574.09	116519.09	116344.31	0.86
*Predictive similarity*							
- Unconstrained across country	−56650.91	21	113343.82	113513.13	113492.13	113425.40	0.86
- Constrained across country	−56660.69	13	113347.38	113452.20	113439.20	113397.89	0.86
*Explanatory similarity*							
- Unconstrained across country	−72421.46	27	144896.92	145115.18	145088.18	145002.38	0.88
- Constrained across country	−72548.36	17	145130.72	145268.15	145251.15	145197.12	0.88
**Multilevel LPA**							
Configural	−13739.54	19	27517.07	27670.67	27651.67	27591.29	0.74
Structural (proportion of L1 profiles)	−13750.02	11	27522.03	27610.96	27599.96	27565.00	0.72
Distributional (proportion of L2 profiles)	−13753.72	10	27527.43	27608.27	27598.27	27564.49	0.72
*Explanatory similarity*							
- Unconstrained across country	−13616.73	20	27273.45	27435.13	27415.13	27351.57	0.76
- Constrained across country	−13665.34	16	27362.68	27492.02	27476.02	27425.18	0.76

*AIC = Akaike Information Criteria. CAIC = Consistent Akaike Information Criteria. BIC = Bayesian Information Criteria. SSA-BIC = sample-size-adjusted Bayesian Information Criteria.*

Teachers corresponding to profile 1 (12% of the sample) reported low barriers to professional development, very low disruptive behavior, high teacher collaboration, high teacher input, and high self-efficacy. This profile was thus labeled *Low-Demand-Flourisher* to reflect this adaptive blend of low job demands and high job and personal resources. Teachers corresponding to profile 2 (17% of the sample) reported low barriers to professional development, average disruptive behavior, high teacher collaboration, high teacher input, and high self-efficacy. This profile was thus labeled *Mixed-Demand-Flourisher* to reflect the mixed blend of low to average job demands, coupled with high job and personal resources. Teachers corresponding to profile 3 (21% of the sample) reported slightly below average barriers to professional development, average disruptive behavior, high teacher collaboration, high teacher input, and average self-efficacy. We labeled this profile *Job-Resourced-Average* to reflect the above average job resources, and average job demands and self-efficacy. Teachers corresponding to profile 4 (15% of the sample) reported average barriers to professional development, average disruptive behavior, average teacher collaboration, average teacher input, and average self-efficacy. We labeled this profile *Balanced-Average* to reflect the matching average levels observed across all demands and resources. Teachers corresponding to profile 5 (34% of the sample) reported high barriers to professional development, high disruptive behavior, low teacher collaboration, low teacher input, and low self-efficacy. We labeled this profile *Struggler* to reflect this blend of high job demands, and low job and personal resources.

We next tested the predictive and explanatory similarity of this solution by including predictors (i.e., gender, teaching experience) and outcomes to this final model of distributional similarity. In terms of predictive similarity, the CAIC, BIC and SSA-BIC decreased when equality constraints across countries were included for the predictive paths (see Table 3), thus supporting the equivalence of these predictions across countries. The results from the multinomial regression paths estimated as part of this model are reported in Table 4. These results first show that Male teachers were less likely to correspond to the Low-Demand-Flourisher and Mixed-Demand-Flourisher profiles than to the Job-Resourced-Average, Balanced-Average, or Struggler profiles. Teachers with more extensive teaching experience were more likely to correspond to the Low-Demand-Flourisher profile than to the Mixed-Demand-Flourisher, Job-Resourced-Average, Balanced-Average, and Struggler profiles. Teachers with more extensive teaching experience were also more likely to correspond to the Mixed-Demand-Flourisher and Job-Resourced-Average profiles than to the Struggler profile. Taken together, male teachers and less experienced teachers were more likely to correspond to the apparently less desirable profiles.

**TABLE 4:** The role of teacher covariates in predicting profile membership in both countries from the single-level LPA.

	*b*	*SE*	*OR*	*b*	*SE*	*OR*
		
	Low-demand-flourisher vs. Mixed-demand-flourisher			Low-demand-flourisher vs. Job-resourced-average		
Gender (F/M)	0.17	0.10	1.18	−0.33**	0.09	0.72
Teaching experience	0.03**	0.01	1.03	0.04**	0.01	1.04

	**Low-Demand-Flourisher vs. Balanced-Average**			**Low-Demand-Flourisher vs. Struggler**		

Gender (F/M)	−0.41**	0.10	0.66	−0.46**	0.09	0.63
Teaching experience	0.04**	0.01	1.04	0.05**	0.01	1.05

	**Mixed-Demand-Flourisher vs. Job-Resourced-Average**			**Mixed-Demand-Flourisher vs. Balanced-Average**		

Gender (F/M)	−0.50**	0.09	0.61	−0.58**	0.10	0.56
Teaching experience	0.01	0.01	1.01	0.01	0.01	1.01

	**Mixed-Demand-Flourisher vs. Struggler**			**Job-Resourced-Average vs. Balanced-Average**		

Gender (F/M)	−0.62**	0.09	0.54	−0.08	0.09	0.92
Teaching experience	0.02**	0.01	1.02	0.01	0.01	1.01

	**Job-Resourced-Average vs. Struggler**			**Balanced-Average vs. Struggler**		

Gender (F/M)	−0.13	0.07	0.88	−0.05	0.08	0.95
Teaching experience	0.01**	0.01	1.01	0.01	0.01	1.01

*p ≤ 0.05; ** p ≤ 0.01; b = multinomial logistic regression coefficient; SE = standard error of the coefficient; OR = odds ratio; For gender, females were coded 0 and males were coded 1.

In terms of explanatory similarity, the CAIC, BIC and SSA-BIC increased when profile-specific outcome levels were constrained to be equal across countries (see Table 3), suggesting that these outcomes associations were not equivalent across countries. To investigate the differences, we compared the means of the outcomes within and across the two countries. For Australia, there were significant differences in means across all profiles for both outcomes (*p <* 0.05). The Mixed-Demand-Flourisher profile displayed the highest levels of job satisfaction (*M* = 3.64) and commitment (*M* = 3.54), followed by the Low-Demand-Flourisher profile (*M* = 3.53 for job satisfaction and *M* = 3.46 for commitment), then by the Job-Resourced-Average profile (*M* = 3.36 and *M* = 3.23), followed by the Balanced-Average profile (*M* = 3.13 and *M* = 3.09), and finally by the Struggler profile (*M* = 2.88 and *M* = 2.89).

For England, all mean comparisons were also statistically significant (*p ≤* 0.05), with one exception. Starting with job satisfaction, the Mixed-Demand-Flourisher (*M* = 3.53) displayed the highest levels, followed by the Low-Demand-Flourisher profile (*M* = 3.41), then by the Job-Resourced-Average profile (*M* = 3.23), followed by the Balanced-Average profile (*M* = 3.06), and finally by the Struggler profile (*M* = 2.59). For occupational commitment, the Mixed-Demand-Flourisher displayed the highest levels (*M* = 3.56), followed by the Low-Demand-Flourisher profile (*M* = 3.44) and then equally by the Job-Resourced-Average (*M* = 3.18) and Balanced-Average (*M* = 3.13) profiles, which did not differ from one another, and finally by the Struggler profile (*M* = 2.75).

Finally, we also tested mean differences within matching profiles across the two countries. All profiles except one from the Australian sample displayed higher levels of job satisfaction when compared with the matching profiles from the English sample (*p <* 0.01). The exception was related to the Balanced-Average profile, which displayed similar levels of job satisfaction in both countries. For occupational commitment, there were no significant differences between countries, with the sole exception of the Struggler profile for which levels of occupational commitment were higher in Australia than in England (*p* <0.01).

### Multilevel LPA

The fit statistics associated with the multilevel solutions including 1 to 8 profiles estimated separately in Australia and England are reported in Table 2 (corresponding elbow plots are reported in the Supplementary Material). In both countries, the solution including two school-level (L2) profiles resulted in the lowest value for the CAIC and BIC. The SSA-BIC was also lowest for the 2-profile solution in England, and although it kept in decreasing up until the 3-profile solution in Australia, the elbow plot displayed a clear flattening after the 2 profile solution. Finally, although the AIC kept on decreasing until the 5-profile solution in Australia and the 4-profile solution in England, this decrease also showed a marked flattening after 2 profiles in both countries. Taken together, these statistical results thus strongly support the 2-profile solution in both countries. Examination of this solution, together with an examination of the adjacent solutions, supported the theoretical value of considering two profiles, but not that of adding a third profile, which did not seem to bring any new information. Accordingly, a solution with 2 school-level profiles was selected as the final solution for both countries.

**FIGURE 3:**
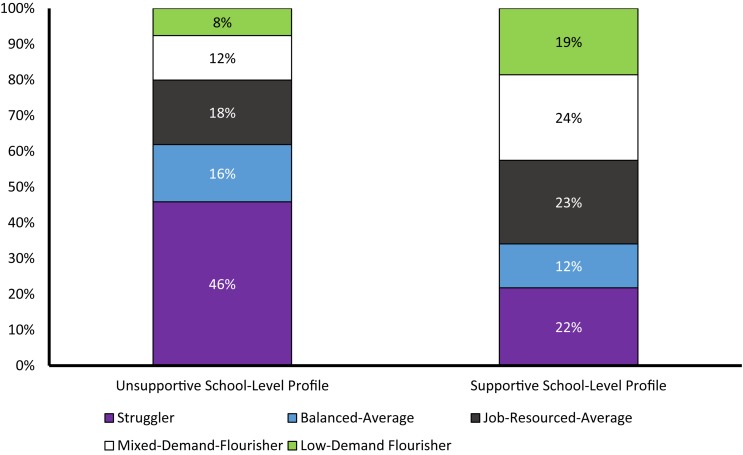
Multilevel LPA results (with L1 and L2 distributional constraints) showing the school-level profiles for both countries.

The results from the L2 tests of profile similarity conducted across countries are reported in Table 3. These results revealed that, each step of the sequence of similarity tests resulted in a decrease in the value of the CAIC, BIC, and SSA-BIC, thus supporting the complete (configural, structural, and distributional) similarity of the solution across countries. A graphical representation of this final 2-profile solution of distributional similarity is presented in Figure 3. Examination of this solution suggested the presence of one Unsupportive school profile (58% of the schools) and one Supportive school profile (42% of the schools). The Unsupportive school profile included a high proportion of members from the Struggler (46%) profile, followed by the Job-Resourced-Average (18%), Balanced-Average (16%), Mixed-Demand-Flourisher (12%), and Low-Demand-Flourisher (8%) profiles. In contrast, the Supportive school profile included a higher proportion of members from the Mixed-Demand-Flourisher (24%) profile, followed by the Job-Resourced-Average (23%), Struggler (22%), Low-Demand-Flourisher (19%), and Balanced-Average (12%) profiles.

We next tested the explanatory similarity of this solution by including outcomes to this final model of distributional similarity. As for the single level models, the CAIC, BIC and SSA-BIC increased when profile-specific outcome levels were constrained to be equal across countries (see Table 3), suggesting that these outcomes associations were not equivalent across countries. To investigate the differences, we compared the school-level means of the outcomes within and across the two countries. For Australia, the Unsupportive school profile displayed significantly lower (*p* < 0.01) school-average job satisfaction (*M* = 3.01) and occupational commitment (*M* = 3.04) than the Supportive school profile (*M* = 3.37 and *M* = 3.27, respectively). The same was true for England, where the Unsupportive school profile also displayed significantly lower (*p* ≤ 0.01) school-average job satisfaction (*M* = 2.79) and occupational commitment (*M* = 2.92) than the Supportive school profile (*M* = 3.16 and *M* = 3.22, respectively). Finally, the two L2-profiles from the Australian sample displayed higher levels of job satisfaction than the matching profiles estimated in the English sample (*p* < 0.01). In addition, the Unsupportive school profile from the Australian sample displayed higher levels of occupational commitment than the matching profile from the English sample (*p* ≤ 0.01), but no differences in occupational commitment were observed for the Supportive school profile across country.

## Discussion

We used person-centered analyses to identify demand-resource profiles among teachers and schools across representative samples from two different countries. In phase one, five teacher-level demand-resources profiles were identified in both the Australian and English samples: the Low-Demand-Flourisher, Mixed-Demand-Flourisher, Job-Resourced-Average, Balanced-Average, and Struggler. Results showed that male teachers and less experienced teachers were more likely to be members of less adaptive profiles in both countries (e.g., the Struggler profile). The profiles were also associated with different levels on both well-being outcomes (highest for the Mixed-Demand-Flourisher profile, lowest for the Struggler profile) in each country. More precise cross-country comparisons showed that profile-specific levels of job satisfaction were higher in the Australian sample than levels observed in the matching English profiles, whereas few differences in occupational commitment were evident.

In phase two, we extended our analyses to the school-level. In both countries, we found evidence of two profiles of schools: a Supportive school profile comprising relatively similar levels of the Mixed-Demand-Flourisher, Job-Resourced-Average, Struggler, and Low-Demand-Flourisher profiles, and an Unsupportive school profile comprising much higher levels of the Struggler profile. For both countries, the Supportive school profile was associated with significantly higher levels of school-average job satisfaction and occupational commitment. Cross-country differences in outcomes levels were also apparent, and are discussed below.

### Findings of Note From the Teacher-Level Results

As noted, five demand-resource profiles were evident at the teacher-level for both Australia and England. Two of these profiles were named flourishers, the Low-Demand-Flourisher and Mixed-Demand-Flourisher, and in combination made up almost one-third of the sample. Given the nature of these two profiles (low or average job demands, high job and personal resources), this is a positive finding. The third profile, the Job-Resourced-Average, represented around one-fifth of the sample. Thus, around 1 in 5 teachers in the sample experienced average job demands and self-efficacy, but simultaneously felt well-supported (above average job resources). The fourth profile, the Balanced-Average, represented 15% of the sample. These teachers appear to experience relatively similar (and average) levels of demands and resources. Finally, the Struggler represented around one-third of each sample. Thus, 1 in 3 teachers in the sample experienced high job demands, low job resources, and low self-efficacy.

Taken together, the findings are important as they provide insight into the groups of teachers that work in schools. The findings are also significant because of the close overlap in the profiles demonstrated across the two countries—which may have occurred given the historical and socio-cultural similarities of the two contexts (e.g., Bulle, 2011). Importantly, there is some commonality between our five profiles and those found in prior research among other types of employees (e.g., Van den Broeck et al., 2012). However, unlike Van den Broeck et al. (2012), we did not find evidence of a low job demands and low job resources profile, nor a high job demands and high job resources profile. At the same time, the Balanced-Average profile (with average demands and resources) seems to exist between these two extremes. Thus, it may be that matching levels of demands and resources are apparent among teachers, just not at such extreme levels as among other employees. This finding might have occurred because demands and resources are often reflective of the broader means available to a school. Schools with high demands are often under-resourced, whereas the reverse is true for schools with low demands (e.g., Muijs et al., 2004). As such, balanced profiles at extreme levels may be less likely to occur among teachers than in other professions. Additional research with different samples is needed to further test this. In future research, it will also be important to model the extent of change over time in profile membership to ascertain the stability of such groupings.

The findings provide a nuanced understanding that complements prior variable-centered results showing that job and personal resources are typically positively correlated. For instance, Collie et al. (2012) found that teacher self-efficacy and input in decision making were positively correlated (*r* = 0.22). Conversely, our findings showed that while personal resources and job resources appeared to be at similar levels for most profiles (i.e., the two Flourishers, the Balanced-Average, the Struggler), they were less aligned for the Job-Resourced-Average profile. Taken together, these findings highlight the merits of person-centered research given that it is able to access the experiences of different subpopulations of teachers.

In both countries, the background characteristics were associated with the profiles in similar ways, showing that male teachers were more likely to correspond to the Job-Resourced-Average, Balanced-Average, or Struggler profiles than to either of the two Flourisher profiles. It is possible this finding occurred because, relative to male teachers, female teachers have been shown to report stronger perceptions of job resources generally (Skaalvik and Skaalvik, 2018), greater teacher collaboration (Ronfeldt et al., 2015), and greater self-efficacy (Klassen and Chiu, 2010). Less experienced teachers were typically more likely to be in the Job-Resourced-Average, Balanced-Average, or Struggler profiles than in either of the two Flourisher profiles. These findings are not surprising. Teaching is a complex job and beginning teachers must navigate this complexity with less knowledge and less first-hand experience to draw upon (Mansfield et al., 2014). It is thus understandable that less experienced teachers tended to appear within profiles characterized by higher demands and lower resources. Of importance, this finding highlights the salience of providing higher levels of support for early career teachers as shown in other research (e.g., De Neve et al., 2015).

Turning to the outcomes, the Mixed-Demand-Flourisher profile displayed the highest levels of job satisfaction and occupational commitment in both countries. This was followed by the Low-Demand-Flourisher profile. These results are understandable given the nature of the two Flourisher profiles, and given that low demands and high resources have been associated with well-being in prior variable-centered research (e.g., Skaalvik and Skaalvik, 2018). The significant differences between the two Flourisher profiles in our results are also interesting and hold potential contributions for theory. Notably, the boosting process in JD-R theory stipulates that resources play an even stronger role in promoting well-being outcomes when demands are high (Bakker and Demerouti, 2017). Perhaps the Mixed-Demand-Flourisher profile displayed more positive outcomes than the Low-Demand-Flourisher profile because with relatively higher levels of disruptive student behavior, the resources available to the Mixed-Demand-Flourisher profile became more important for their well-being. Conversely, the Low-Demand-Flourisher profile experienced low job demands and thus the resources were perhaps less relevant and then less salient for well-being (Bakker et al., 2007). What is interesting about these results is they suggest that average levels of job demands are not necessarily problematic. As along as demands are outweighed by resources, teachers may still experience high levels of well-being. Future research is needed to test whether this suggestion replicates with other samples.

Moving along to the other profiles, the Job-Resourced-Average profile typically displayed the third highest levels of the well-being outcomes, followed by the Balanced-Average profile. In comparing these two profiles, the major differences occurred in job resources, which underscores prior research on the importance of contextual supports for teachers (e.g., Lee and Nie, 2014; Desrumaux et al., 2015). The Job-Resourced-Average profile had access to greater job resources, which may have meant a boost to their well-being. In contrast, the relatively equal levels of demands and resources for the Balanced-Average profile may have meant the boosting effect was not as evident (because this profile did not have particularly high resources to drawn upon). Future research is needed to disentangle these results and see if they are replicated.

Finally, the Struggler profile displayed the lowest outcomes. Alongside the mismatch between (high) demands and (low) resources that this profile experienced, low collaboration and input in decision-making may mean that teachers in this profile experience a reduced sense of autonomy (e.g., Ryan and Deci, 2017) and lower professional fit at work (e.g., Kristof, 1996). Both a sense of autonomy and professional fit have been identified as important for teachers’ job satisfaction (e.g., Collie et al., 2016). Moreover, if teachers’ feel their professional growth is not being fostered (e.g., via barriers to professional development), they typically have less desire to remain in the profession (e.g., Ford et al., 2019).

Taken together, the findings involving the outcomes complement knowledge gained from prior variable-centered research by providing a more nuanced understanding of the associations that demands and resources have with outcomes related to well-being. Prior research has clearly documented that job demands are typically negatively associated with well-being, whereas the reverse is true for resources (e.g., Skaalvik and Skaalvik, 2018). In a complementary manner, our results highlight how varying combinations of demands and resources are also related to differences in levels of well-being. This knowledge provides a clearer picture of the simultaneous role of multiple factors in teachers’ work.

In terms of cross-country comparisons, significantly higher levels of job satisfaction were evident in most of the profiles from the Australian sample when compared with the matching profiles from the English sample. For occupational commitment, only the Struggler profile displayed higher levels in the Australian sample than in the English sample. Additional research is needed to understand precisely why these findings occurred, but it may be related to increases in compliance and reductions in professional autonomy that have been documented in England over the past decade (e.g., Adams, 2017)—such working conditions are known to be unsatisfying for teachers (e.g., Skaalvik and Skaalvik, 2014). Perhaps occupational commitment was not significantly lower among the teachers from England (except for the Struggler profile) because this construct is reflective of teachers’ longer-term motives for being in the teaching profession (e.g., helping students), which are somewhat more distal from day-to-day working conditions. Given that job satisfaction is associated with lower motivation to quit the profession (Skaalvik and Skaalvik, 2017) and lower burnout (Malinen and Savolainen, 2016), the low levels of this outcome among the English sample might have longer-term ramifications. More precisely, even though the English teachers were committed to the profession, they may not be functioning as effectively as possible at work due to their lower job satisfaction and this may result in negative outcomes later. Going forward, it will be important for longitudinal research to explore this.

### Findings of Note From the School-Level Results

As noted earlier, large scale datasets from surveys like TALIS enable insights into phenomena at a school-level that are often inaccessible with smaller datasets. In our study, two profiles were evident at the school-level in both countries. The first we called the Unsupportive school profile and comprised 58% of participating teachers. The second was the Supportive school profile (comprising around 42% of participating teachers), and included a substantially greater proportion of the two Flourisher profiles, and a smaller proportion of the Struggler profile than the Unsupportive school profile. The higher proportion of schools corresponding to the Unsupportive school profile is in accord with the growing attention toward the escalating demands faced by many teachers and schools in Australia and England. Indeed, there is growing attention to teachers’ workload, burnout, and attrition from government working groups (UK Department for Education, 2018; Parliament of Australia, 2019) and not-for-profit organizations (e.g., Education Support Partnership, 2018) in both countries.

Notably, our results are some of the first to examine demand-resource profiles at the school-level among teachers, and the first to test profile similarity at the school-level across country. Our findings are important because limited research has examined multilevel associations using JD-R theory (Bakker and Demerouti, 2018). Moreover, whereas multilevel variable-centered research reveals knowledge about how variables are associated at the school-level, the current study identifies the types of teachers that predominate in different schools. By revealing types of schools, our findings add to knowledge about particular variables that might be important at the school level (e.g., school climate; Klassen et al., 2012). Taken together, our teacher- and school-level results provide important knowledge relevant for practice and policy on teacher well-being. More precisely, by considering all findings simultaneously, it is apparent that efforts to address not only the individual, but also the school, are warranted (further details below). Indeed, attending to one level (teacher or school), but not to both levels simultaneously, might result in efforts that are less effective in the longer term.

Turning to the outcomes, the Supportive school profile was associated with significantly higher levels of school-average job satisfaction and occupational commitment. This finding contributes to the literature by highlighting that the particular combination of teacher types within a school is associated with school-average teacher well-being. It is possible that this finding occurred because the Supportive school profile was characterized by a higher proportion of the two Flourisher profiles, which had more positive outcome levels. When teachers’ resources outweigh their demands, then teachers are better equipped to undertake their work and are likely more satisfied with their jobs and committed to their profession (e.g., Simbula et al., 2012; Collie et al., 2015; Skaalvik and Skaalvik, 2018). It is also possible that social contagion occurs in schools with more satisfied and committed teachers, helping to further promote these outcomes—though additional research is needed to examine this. Of note, these findings suggest that there may be merit in promoting school-wide approaches to addressing teacher well-being (in addition to efforts focused on the individual; discussed below).

### Implications for Practice

Because the current study is one of the first to consider demand-resource profiles among teachers, we emphasize that more research is needed to ascertain the generalizability of our findings. Nonetheless, we do provide some tentative suggestions for practice. Notably, a key contribution of person-centered research is that it allows implications for practice that are targeted more closely to the needs of particular subpopulations of teachers and schools. For example, teachers in the Flourisher profiles would likely benefit from efforts to further boost (or at least maintain) their access to resources. For the Struggler profile, efforts may want to focus on reducing demands and increasing resources. Turning to the school-level, efforts targeted at the Unsupportive school profile may focus on school-wide efforts to boost resources and reduce job demands, whereas efforts focused on the Supportive school profile may focus on further boosting job and personal resources.

In terms of practices that can help boost resources and reduce demands, reducing barriers to professional development is likely important. As noted earlier, barriers include budget constraints, availability, support from leadership, and time (e.g., Kwakman, 2003). Efforts by schools to reduce each of these barriers will support teachers in accessing the professional development and learning they require. For example, in rural and remote areas, schools might focus more on online professional learning (e.g., see Broadley, 2010). By reducing barriers to professional development, teachers may gain more access to additional strategies and learning opportunities that are relevant to the other demands and resources we examined. For example, professional learning via reflection can help to improve teachers’ capacity to effectively navigate disruptive behavior and improve teacher-student relationships (e.g., Spilt et al., 2012).

In terms of resources, engagement in professional learning communities and instructional rounds (observing other teachers) are two effective methods for encouraging teacher collaboration (e.g., Durksen et al., 2017) and boosting teacher self-efficacy (e.g., Chong and Kong, 2012). Finally, a growing body of research has shown that input in decision-making is important for teachers (e.g., Klassen et al., 2012). School leaders can promote teachers’ input by listening to teachers’ needs, attempting to understand issues from teachers’ perspectives, and seeking teachers’ suggestions for decisions that are made (e.g., Ware and Kitsantas, 2011).

### Limitations and Future Directions

Consideration of several limitations is important in interpreting findings from the current study. First, the use of TALIS 2013 provides significant strengths (e.g., nationally representative samples, matching teacher and school-level data). Nonetheless, this type of data does come with some limitations. In particular, our study was cross-sectional in nature, which means that we are unable to ascertain causal ordering between the profiles and the outcomes, nor whether teachers’ profile membership fluctuates over time. Longitudinal modeling (e.g., latent transition analysis) will be an important avenue to explore in future. Second, we focused on five demands and resources. As noted earlier, our selection of factors was firmly based in JD-R theory, conceptual reasoning, and prior empirical research. Nonetheless, in future it will be important to consider a different range of factors to see what else is salient in teachers’ experiences. Third, our study employed data from teachers. Of course, teachers’ perceptions are essential given that it is their interpretations that may influence their well-being outcomes. Nonetheless, in future it will be interesting to consider other markers of demands and resources to see how perceptions of demands and resources align with measures taken from other informants (e.g., school principals). Fourth, our study was conducted among teachers from Australia and England. Examining the extent to which similar profiles can identified, or not, in other countries (including non-English speaking countries) should be an important upcoming research focus. As noted earlier, teachers’ experiences of demands and resources in Australia and England are mirrored in many other countries (e.g., Skaalvik and Skaalvik, 2018). Nonetheless, more evidence is needed before it is possible to argue that these results apply to broader contexts.

## Conclusion

The aim of the current study was to establish whether different profiles of teachers could be identified based on their experiences of demands and resources, and, if so, to ascertain which profiles are more aligned with well-being. We conducted our examination at the teacher- and school-level among teachers from Australia and England. Findings revealed five teacher profiles that were similar across the two countries: the Low-Demand-Flourisher, Mixed-Demand-Flourisher, Job-Resourced-Average, Balanced-Average, and Struggler. Notably, the profiles differed in relation to two well-being outcomes, with the Mixed-Demand-Flourisher typically evincing the highest levels of job satisfaction and occupational commitment. Two school-level profiles that were similar in both countries were identified based on the prevalence of the five teacher profiles: the Unsupportive and Supportive school profiles. Of note, the Supportive school profile was associated with higher school-average teacher job satisfaction and occupational commitment. Taken together, the findings yield novel understanding about different subgroups of teachers and schools, and hold implications for practice at the teacher- and school-level.

## Data Availability Statement

The datasets used in our research are available from the OECD.

## Ethics Statement

Ethical review and approval was not required for the study on human participants in accordance with the local legislation and institutional requirements. Written informed consent for participation was not required for this study in accordance with the national legislation and the institutional requirements.

## Author Contributions

All authors listed have made a substantial, direct and intellectual contribution to the work, and approved it for publication.

## Conflict of Interest

The authors declare that the research was conducted in the absence of any commercial or financial relationships that could be construed as a potential conflict of interest.

## Footnotes

1 Coefficient omega was calculated using factor loadings from congeneric single-level or multilevel CFAs calculated separately for the two countries.
