# Supplementary material for: A Multilevel Person-Centered Examination of Teachers’ Workplace Demands and Resources: Links With Work-Related Well-Being
Source: Front Psychol. 2020 Apr 8;11:626. doi: 10.3389/fpsyg.2020.00626 (PMC7156640; doi:10.3389/fpsyg.2020.00626)
Supplement: Supplementary file 1 [file Data_Sheet_1.docx]

**Online Supplemental Materials for**

**A Multilevel Person-centered Examination of Teachers’ Workplace Demands and Resources: Links with Work-Related Well-being**

1. Table S1 contains latent correlations from the CFAs for Australia and England, which involved the two covariates, the 5 demands and resources (where self-efficacy was modelled as a higher-order factor), and the two outcomes. The demands, resources, and outcomes were estimated as latent factors (using the items indicated in Measures) and the covariates were estimated with the loading set to 1 and the residual to 0. Fit indices indicated adequate fit for both countries. For Australia, χ^2^(557) = 4820.62, *p*<.001, RMSEA=.034, CFI=.92, and TLI = .91. For England, χ^2^(557) = 2687.44, *p*<.001, RMSEA=.039, CFI=.92, and TLI = .91.
2. Figure S1 shows elbow plots from the single-level LPA for Australia (Figure S1a) and England (Figure S1b).
3. Figure S2 shows elbow plots from the multilevel LPA for Australia (Figure S2a) and England (Figure S2b).
4. Table S2 displays results from the single-level LPA: the indicator variable means and variances (with 95% confidence intervals).
5. Table S3 shows the item numbers from TALIS 2013 (OECD, 2014) used to assess each construct in the study.
6. M*plus* Input Syntax: Standardizing L1 and L2 Weights Separately for Each Country
7. M*plus* Input Syntax: Manual 3-Step Process (First Step) – Saving Start Values for Each Country
8. M*plus* Input Syntax: Manual 3-Step Process (Third Step) – Multilevel LPA with Start Values
9. M*plus* Input Syntax for L2 Profile Similarity Tests Step 1: L2 Configural Similarity
10. M*plus* Input Syntax for L2 Profile Similarity Tests Step 2: L2 Structural Similarity
11. M*plus* Input Syntax for L2 Profile Similarity Tests Step 3: L2 Distributional Similarity
12. M*plus* Input Syntax for L2 Profile Similarity Tests Step 4a: L2 Explanatory Similarity with Outcomes Freely Estimated Across Countries
13. M*plus* Input Syntax for L2 Profile Similarity Tests Step 4b: L2 Explanatory Similarity with Outcomes Constrained to Equality Across Countries

Table S1

*Latent Correlations Among Variables at the Teacher-Level for Australia and England*

|  | 1. | 2. | 3. | 4. | 5. | 6. | 7. | 8. |
| --- | --- | --- | --- | --- | --- | --- | --- | --- |
| *Australian sample* |  |  |  |  |  |  |  |  |
| 1. Gender |  |  |  |  |  |  |  |  |
| 1. Teacher experience | .07** |  |  |  |  |  |  |  |
| 1. Barriers to PL | .09** | -.01 |  |  |  |  |  |  |
| 1. Disruptive student behavior | .05** | -.19** | .22** |  |  |  |  |  |
| 1. Teacher collaboration | -.14** | -.03 | -.13** | -.10** |  |  |  |  |
| 1. Teacher input | -.01 | .02 | -.36** | -.13** | .22** |  |  |  |
| 1. Teacher self-efficacy | -.13** | .14** | -.17** | -.34** | .29** | .13** |  |  |
| 1. Job satisfaction | -.03 | .02 | -.41** | -.30** | .23** | .58** | .26** |  |
| 1. Occ. commitment | -.10** | -.01 | -.35** | -.23** | .16** | .27** | .27** | .67** |
| *English sample* |  |  |  |  |  |  |  |  |
| 1. Gender |  |  |  |  |  |  |  |  |
| 1. Teacher experience | .04 |  |  |  |  |  |  |  |
| 1. Barriers to PL | -.01 | -.05* |  |  |  |  |  |  |
| 1. Disruptive student behavior | -.04 | -.21** | .24** |  |  |  |  |  |
| 1. Teacher collaboration | -.09** | -.04* | -.25** | -.10** |  |  |  |  |
| 1. Teacher input | .02 | .05 | -.49** | -.16** | .34** |  |  |  |
| 1. Teacher self-efficacy | -.04 | .09** | -.17** | -.33** | .30** | .19** |  |  |
| 1. Job satisfaction | .04* | .01 | -.48** | -.29** | .29** | .69** | .32** |  |
| 1. Occ. commitment | -.01 | -.03 | -.36** | -.23** | .20** | .33** | .28** | .68** |

*Note.* Teacher gender was coded 0 for female, 1 for male. Barriers to PL = Barriers to professional learning. Occ. commitment = Occupational commitment.

* *p* < .05, ** *p* < .01.

Figure S1

*Elbow Plots for Single-level LPA Involving Australia (Figure S1a) and England (Figure S1b)*

a)

b)

Figure S2

*Elbow Plots for Multilevel LPA Involving Australia (Figure S2a) and England (Figure S2b)*

a)

b)

Table S2

*Teacher-Level Latent Profile Analysis Solution (Means and Variances) of Indicator Variables from Distributional Similarity Model*

|  | Profile indicator variables | | | | | | | | | | | | | |
| --- | --- | --- | --- | --- | --- | --- | --- | --- | --- | --- | --- | --- | --- | --- |
|  | Barriers to professional development | |  | Disruptive student behavior | |  | Teacher collaboration | |  | Teacher input | |  | Teacher self-efficacy | |
|  | *M*  (95% CI) | Variance  (95% CI) |  | *M*  (95% CI) | Variance  (95% CI) |  | *M*  (95% CI) | Variance  (95% CI) |  | *M*  (95% CI) | Variance  (95% CI) |  | *M*  (95% CI) | Variance  (95% CI) |
| Low-Demand-Flourisher | -0.52  (-0.61, -0.44) | 1.04  (0.95, 1.13) |  | -1.39  (-1.40, -1.39) | 0.01  (0.01, 0.01) |  | 0.36  (0.27, 0.45) | 0.60  (0.46, 0.73) |  | 0.27  (0.19, 0.36) | 1.05  (0.94, 1.15) |  | 0.89  (0.80, 0.98) | 0.36  (0.29, 0.44) |
| Mixed-Demand-Flourisher | -0.65  (-0.78, -0.52) | 0.97  (0.86, 1.07) |  | -0.13  (-0.21, -0.05) | 0.55  (0.45, 0.65) |  | 0.71  (0.64, 0.78) | 0.18  (0.15, 0.22) |  | 0.56  (0.43, 0.68) | 0.87  (0.77, 0.98) |  | 0.61  (0.51, 0.71) | 0.52  (0.44, 0.60) |
| Job-Resourced-Average | -0.19  (-0.26, -0.12) | 0.38  (0.32, 0.45) |  | 0.09  (0.03, 0.15) | 0.82  (0.75, 0.88) |  | 0.24  (0.16, 0.31) | 0.52  (0.43, 0.60) |  | 0.51  (0.51, 0.52) | 0.01  (0.01, 0.01) |  | -0.07  (-0.14, 0.01) | 0.86  (0.80, 0.92) |
| Balanced-Average | 0.10  (0.02, 0.19) | 0.70  (0.61, 0.79) |  | 0.02  (0.02, 0.03) | 0.01  (0.01, 0.01) |  | -0.08  (-0.18, 0.02) | 0.74  (0.64, 0.85) |  | -0.10  (-0.19, -0.01) | 0.80  (0.70, 0.90) |  | -0.15  (-0.24, -0.06) | 0.53  (0.48, 0.58) |
| Struggler | 0.59  (0.53, 0.65) | 0.84  (0.78, 0.90) |  | 0.50  (0.43, 0.57) | 1.16  (1.09, 1.22) |  | -0.59  (-0.66, -0.53) | 1.28  (1.20, 1.35) |  | -0.66  (-0.72, -0.59) | 0.97  (0.92, 1.03) |  | -0.52  (-0.58, -0.46) | 1.01  (0.94, 1.07) |

*Note.* 95% CI = 95% Confidence Interval.

Table S3

*List of Items from TALIS 2013*

|  | Item Number from TALIS 2013 (OECD, 2014) |
| --- | --- |
| *Job Demands* |  |
| Barriers to professional development | TT2G27B – G |
| Disruptive student behavior | TT2G41A, C, and D |
| *Job Resources* |  |
| Teacher collaboration | TT2G33D – F |
| Teacher input | TT2G44A, D, and E |
| *Personal Resource* |  |
| Teacher self-efficacy | TT2G34A – L for the higher-order construct used in modelling  For the first-order constructs:   - Self-efficacy for classroom management: TT2G34D, F, H, I - Self-efficacy for instruction: TT2G34C, J, K, L - Self-efficacy for student engagement: TT2G34A, B, E, G) |
| *Outcomes* |  |
| Job satisfaction | TT2G46E, G, J |
| Occupational commitment | TT2G46A, B, D, and F |

**Mplus Input Syntax: Standardizing L1 and L2 Weights Separately for Each Country**

DATA: FILE IS "fscores.dat";

*!This input standardizes the weights across the two countries so that participants are appropriately grouped to each country. Take the saved data from here and use in the subsequent analyses. This step is only needed if weights are being used.*

VARIABLE: NAMES ARE TT2G01 TT2G05B JOBSAT OCCCOMM SCHWGT PLBARRS PLBARRS_SE SMISBEH SMISBEH_SE TCOLLAB TCOLLAB_SE AUTONOMY AUTONOMY_SE TEFFENG TEFFENG_SE TEFFMAN TEFFMAN_SE TEFFINS TEFFINS_SE TEFF TEFF_SE TCHWGT IDCNTRY SCHLID;

USEVARIABLES = PLBARRS SMISBEH TCOLLAB AUTONOMY TEFF;

GROUPING = IDCNTRY (36=AUS 926=ENG);

WITHIN = PLBARRS SMISBEH TCOLLAB AUTONOMY TEFF;

CLUSTER = SCHLID;

MISSING ARE *;

weight=TCHWGT;

wtscale = cluster;

bweight=SCHWGT;

bwtscale = sample;

AUXILIARY ARE TT2G01 TT2G05B JOBSAT OCCCOMM;

DEFINE:

STANDARDIZE PLBARRS SMISBEH TCOLLAB AUTONOMY TEFF;

ANALYSIS: TYPE = TWOLEVEL;

SAVEDATA: FILE IS MG-MLPA.DAT;

**Mplus Input Syntax: Manual 3-Step Process (First Step) – Saving Start Values for Each Country**

*! This input is an example of the first step in the manual 3-step process where start values are saved. This input should be run for each group separately (e.g., each country). Then the logits for the classification probabilities are taken from the output, which is the second step (use “Logits for the Classification Probabilities for the Most Likely Latent Class Membership [Column] by Latent Class [Row]”) in the third step with the saved data (see next section). See Litalien et al., 2019 and Morin & Litalien, 2017 for further details.*

DATA: FILE IS "MG-MLPA.dat";

VARIABLE: NAMES ARE PLBARRS SMISBEH TCOLLAB AUTONOMY TEFF TT2G01 TT2G05B

JOBSAT OCCCOMM TCHWGT SCHWGT IDCNTRY SCHLID;

USEVARIABLES = PLBARRS SMISBEH TCOLLAB AUTONOMY TEFF;

CLASSES = c (5);

CLUSTER = SCHLID;

MISSING ARE *;

weight=TCHWGT;

SUBPOP ARE (IDCNTRY EQ 36);

AUXILIARY = TT2G01 TT2G05B JOBSAT OCCCOMM SCHWGT IDCNTRY;

DEFINE:

STANDARDIZE PLBARRS SMISBEH TCOLLAB AUTONOMY TEFF;

ANALYSIS:

TYPE = MIXTURE COMPLEX;

STARTS = 0;

MODEL:

%OVERALL%

*!The lines below specify that the means for the categorical latent variables for each profile are constrained to the values from the most similar model from the level 1 (L1) profile similarity tests (i.e., L1-distributional similarity in our study).*

[ c#1@-0.54796 ];

[ c#2@-0.22088 ];

[ c#3@0.47137 ];

[ c#4@-0.38942 ];

*!Below, the means and variances for the different profiles are set to the parameters estimated in the L1-distributional similarity tests. The labels (m1, m2) in parentheses can be deleted (given that the parameters are fixed) but they can also be retained in the analyses for greater simplicity (they are automatically included in Mplus SVALUES section).*

%C#1%

[ plbarrs@-0.52365 ] (m1);

[ smisbeh@-1.39406 ] (m2);

[ tcollab@0.35766 ] (m3);

[ autonomy@0.27375 ] (m4);

[ teff@0.88642 ] (m5);

plbarrs@1.04080 (v1);

smisbeh@0.00077 (v2);

tcollab@0.59714 (v3);

autonomy@1.04542 (v4);

teff@0.36301 (v5);

%C#2%

[ plbarrs@-0.64772 ] (m7);

[ smisbeh@-0.12982 ] (m8);

[ tcollab@0.70789 ] (m9);

[ autonomy@0.55578 ] (m10);

[ teff@0.61183 ] (m11);

plbarrs@0.96463 (v11);

smisbeh@0.54752 (v12);

tcollab@0.18441 (v13);

autonomy@0.87361 (v14);

teff@0.51759 (v15);

%C#3%

[ plbarrs@0.58550 ] (m13);

[ smisbeh@0.50308 ] (m14);

[ tcollab@-0.59440 ] (m15);

[ autonomy@-0.65600 ] (m16);

[ teff@-0.51971 ] (m17);

plbarrs@0.84026 (v21);

smisbeh@1.15504 (v22);

tcollab@1.27656 (v23);

autonomy@0.97322 (v24);

teff@1.00460 (v25);

%C#4%

[ plbarrs@0.10435 ] (m19);

[ smisbeh@0.01967 ] (m20);

[ tcollab@-0.08391 ] (m21);

[ autonomy@-0.09921 ] (m22);

[ teff@-0.15022 ] (m23);

plbarrs@0.69641 (v31);

smisbeh@0.00106 (v32);

tcollab@0.74342 (v33);

autonomy@0.80099 (v34);

teff@0.52838 (v35);

%C#5%

[ plbarrs@-0.18668 ] (m25);

[ smisbeh@0.09069 ] (m26);

[ tcollab@0.23481 ] (m27);

[ autonomy@0.51434 ] (m28);

[ teff@-0.06860 ] (m29);

plbarrs@0.37999 (v36);

smisbeh@0.81779 (v37);

tcollab@0.51586 (v38);

autonomy@0.00113 (v39);

teff@0.86006 (v40);

OUTPUT: TECH1;

SAVEDATA: file=c36.dat; save=cprob;

*!The new .dat files for every country involved in the analyses must then be merged into a combined data file. We called this globaldat.csv, and this is used in all subsequent analyses*

**Mplus Input Syntax: Manual 3-Step Process (Third Step) – Multilevel LPA with Start Values**

*!This input is an example of a multilevel LPA (ML-LPA) model that uses the saved data, logits for the classification probabilities, and model class assignment from the second step of the Manual 3-Step process. This is the third step of the Manual 3-Step process. See Litalien et al., 2019 and Morin & Litalien, 2017 for further details about the Manual 3-Step process. We used this step to run ML-LPA in each country separately.*

DATA: FILE IS "GLOBALDAT.csv";

VARIABLE: NAMES ARE PLBARRS SMISBEH TCOLLAB AUTONOMY TEFF TT2G01 TT2G05B

JOBSAT OCCCOMM SCHWGT IDCNTRY CPROB1 CPROB2 CPROB3 CPROB4 CPROB5

CL TCHWGT SCHLID;

*!CL is the multinomial model class assignment determined from the second step of the Manual 3-Step process. CW and CB respectively refer to the L1 and L2 latent profiles.*

USEVARIABLES = CL;

NOMINAL = CL;

CLASSES = CB (2) CW (5);

WITHIN = CL;

BETWEEN = CB;

CLUSTER = SCHLID;

MISSING ARE *;

USEOBS ARE (IDCNTRY EQ 36);

weight=TCHWGT;

wtscale = cluster;

bweight=SCHWGT;

bwtscale = sample;

ANALYSIS:

TYPE = MIXTURE TWOLEVEL;

STARTS = 6000 500 100;

STITERATIONS = 1000;

MODEL:

%WITHIN%

%OVERALL%

%BETWEEN%

%OVERALL%

*! This line specifies that the Level 2 (L2) profiles are defined based on the relative frequency of the Level 1 (L1) profiles.*

CW ON CB;

MODEL CW:

%WITHIN%

*!The teacher-level profiles are set to the values (i.e., logits for the classification probabilities) computed in the second step*

%CW#1%

[CL#1@ 4];

[CL#2@ -1.196];

[CL#3@ 0.12];

[CL#4@ -9.772];

%CW#2%

[CL#1@ -1.079];

[CL#2@ 2.401];

[CL#3@ 0.492];

[CL#4@ -0.277];

%CW#3%

[CL#1@ -1.152];

[CL#2@ 0.529];

[CL#3@ 3.512];

[CL#4@ 0.362];

%CW#4%

[CL#1@ -11.599];

[CL#2@ -1.663];

[CL#3@ -1.22];

[CL#4@ 2.04];

%CW#5%

[CL#1@ -3.783];

[CL#2@ -4.263];

[CL#3@ -3.806];

[CL#4@ -3.587];

OUTPUT: TECH1 SVALUES;

**Mplus Input Syntax for L2 Profile Similarity Tests Step 1: L2-Configural Similarity**

DATA: FILE IS "GLOBALDAT.csv";

VARIABLE: NAMES ARE PLBARRS SMISBEH TCOLLAB AUTONOMY TEFF TT2G01 TT2G05B

JOBSAT OCCCOMM SCHWGT IDCNTRY CPROB1 CPROB2 CPROB3 CPROB4 CPROB5

CL TCHWGT SCHLID;

USEVARIABLES = CL;

NOMINAL = CL;

KNOWNCLASS = cg (IDCNTRY = 36 IDCNTRY = 926);

CLASSES = CG (2) CB (2) CW (5);

WITHIN = CL;

BETWEEN = CG CB;

CLUSTER = SCHLID;

MISSING ARE *;

weight=TCHWGT;

wtscale = cluster;

bweight=SCHWGT;

bwtscale = sample;

ANALYSIS:

TYPE = MIXTURE TWOLEVEL;

STARTS = 10000 1000 200;

STITERATIONS = 1000;

MODEL:

%WITHIN%

%OVERALL%

%BETWEEN%

%OVERALL%

*! This line specifies that the Level 2 (L2) profiles are defined based on the relative frequency of the Level 1 (L1) profiles. The @0 is needed in order to allow free estimation of the CW on CB paths within each country in model CG below.*

CW ON CB@0;

*!This line indicates that class sizes (class probabilities) are freely estimated in all groups (i.e., countries)*

CB ON CG;

*!This path is necessary to account for the fact that we assume that the CB -> CW path is moderated by CG.*

CW on CG;

MODEL CG.CW:

%WITHIN%

*!The start values below are those from the third step of the Manual 3-Step Process above.*

*!Group 1 (Australia)*

%cg#1.cw#1%

[CL#1@ 4];

[CL#2@ -1.196];

[CL#3@ 0.12];

[CL#4@ -9.772];

%cg#1.cw#2%

[CL#1@ -1.079];

[CL#2@ 2.401];

[CL#3@ 0.492];

[CL#4@ -0.277];

%cg#1.cw#3%

[CL#1@ -1.152];

[CL#2@ 0.529];

[CL#3@ 3.512];

[CL#4@ 0.362];

%cg#1.cw#4%

[CL#1@ -11.599];

[CL#2@ -1.663];

[CL#3@ -1.22];

[CL#4@ 2.04];

%cg#1.cw#5%

[CL#1@ -3.783];

[CL#2@ -4.263];

[CL#3@ -3.806];

[CL#4@ -3.587];

*! Group 2 (England)*

%cg#2.cw#1%

[CL#1@ 3.978];

[CL#2@ 0.292];

[CL#3@ -0.225];

[CL#4@ -9.78];

%cg#2.cw#2%

[CL#1@ -0.349];

[CL#2@ 2.546];

[CL#3@ 0.241];

[CL#4@ -0.475];

%cg#2.cw#3%

[CL#1@ -0.922];

[CL#2@ 0.948];

[CL#3@ 3.632];

[CL#4@ 0.666];

%cg#2.cw#4%

[CL#1@ -11.307];

[CL#2@ -1.176];

[CL#3@ -0.5];

[CL#4@ 2.339];

%cg#2.cw#5%

[CL#1@ -3.398];

[CL#2@ -3.577];

[CL#3@ -4.275];

[CL#4@ -3.691];

*!These lines allow the free estimation of the CW on CB paths within each country.*

MODEL CG:

%BETWEEN%

%cg#1%

cw#1 ON cb#1;

cw#2 ON cb#1;

cw#3 ON cb#1;

cw#4 ON cb#1;

%cg#2%

cw#1 ON cb#1;

cw#2 ON cb#1;

cw#3 ON cb#1;

cw#4 ON cb#1;

OUTPUT: STDYX CINTERVAL SVALUES RESIDUAL TECH1 TECH7;

**Mplus Input Syntax for L2 Profile Similarity Tests Step 2: L2-Structural Similarity**

DATA: FILE IS "GLOBALDAT.csv";

VARIABLE: NAMES ARE PLBARRS SMISBEH TCOLLAB AUTONOMY TEFF TT2G01 TT2G05B

JOBSAT OCCCOMM SCHWGT IDCNTRY CPROB1 CPROB2 CPROB3 CPROB4 CPROB5

CL TCHWGT SCHLID;

USEVARIABLES = CL;

NOMINAL = CL;

KNOWNCLASS = cg (IDCNTRY = 36 IDCNTRY = 926);

CLASSES = CG (2) CB (2) CW (5);

WITHIN = CL;

BETWEEN = CG CB;

CLUSTER = SCHLID;

MISSING ARE *;

weight=TCHWGT;

wtscale = cluster;

bweight=SCHWGT;

bwtscale = sample;

ANALYSIS:

TYPE = MIXTURE TWOLEVEL;

STARTS = 10000 1000 200;

STITERATIONS = 1000;

MODEL:

%WITHIN%

%OVERALL%

%BETWEEN%

%OVERALL%

*!By default (i.e., without @0), this is equal across countries (CG)*

CW ON CB;

CB ON CG;

MODEL CG.CW:

%WITHIN%

*!Group 1 (Australia)*

%cg#1.cw#1%

[CL#1@ 4];

[CL#2@ -1.196];

[CL#3@ 0.12];

[CL#4@ -9.772];

%cg#1.cw#2%

[CL#1@ -1.079];

[CL#2@ 2.401];

[CL#3@ 0.492];

[CL#4@ -0.277];

%cg#1.cw#3%

[CL#1@ -1.152];

[CL#2@ 0.529];

[CL#3@ 3.512];

[CL#4@ 0.362];

%cg#1.cw#4%

[CL#1@ -11.599];

[CL#2@ -1.663];

[CL#3@ -1.22];

[CL#4@ 2.04];

%cg#1.cw#5%

[CL#1@ -3.783];

[CL#2@ -4.263];

[CL#3@ -3.806];

[CL#4@ -3.587];

*! Group 2 (England)*

%cg#2.cw#1%

[CL#1@ 3.978];

[CL#2@ 0.292];

[CL#3@ -0.225];

[CL#4@ -9.78];

%cg#2.cw#2%

[CL#1@ -0.349];

[CL#2@ 2.546];

[CL#3@ 0.241];

[CL#4@ -0.475];

%cg#2.cw#3%

[CL#1@ -0.922];

[CL#2@ 0.948];

[CL#3@ 3.632];

[CL#4@ 0.666];

%cg#2.cw#4%

[CL#1@ -11.307];

[CL#2@ -1.176];

[CL#3@ -0.5];

[CL#4@ 2.339];

%cg#2.cw#5%

[CL#1@ -3.398];

[CL#2@ -3.577];

[CL#3@ -4.275];

[CL#4@ -3.691];

OUTPUT: STDYX CINTERVAL SVALUES RESIDUAL TECH1 TECH7;

**Mplus Input Syntax for L2 Profile Similarity Tests Step 3: L2-Distributional Similarity**

DATA: FILE IS "GLOBALDAT.csv";

VARIABLE: NAMES ARE PLBARRS SMISBEH TCOLLAB AUTONOMY TEFF TT2G01 TT2G05B

JOBSAT OCCCOMM SCHWGT IDCNTRY CPROB1 CPROB2 CPROB3 CPROB4 CPROB5

CL TCHWGT SCHLID;

USEVARIABLES = CL;

NOMINAL = CL;

KNOWNCLASS = cg (IDCNTRY = 36 IDCNTRY = 926);

CLASSES = CG (2) CB (2) CW (5);

WITHIN = CL;

BETWEEN = CG CB;

CLUSTER = SCHLID;

MISSING ARE *;

weight=TCHWGT;

wtscale = cluster;

bweight=SCHWGT;

bwtscale = sample;

ANALYSIS:

TYPE = MIXTURE TWOLEVEL;

STARTS = 10000 1000 200;

STITERATIONS = 1000;

MODEL:

%WITHIN%

%OVERALL%

%BETWEEN%

%OVERALL%

CW ON CB;

!CB ON CG; *!Removing this line means that class probabilities are constrained for like L2 profiles across the two groups*

MODEL CG.CW:

%WITHIN%

*!Group 1 (Australia)*

%cg#1.cw#1%

[CL#1@ 4];

[CL#2@ -1.196];

[CL#3@ 0.12];

[CL#4@ -9.772];

%cg#1.cw#2%

[CL#1@ -1.079];

[CL#2@ 2.401];

[CL#3@ 0.492];

[CL#4@ -0.277];

%cg#1.cw#3%

[CL#1@ -1.152];

[CL#2@ 0.529];

[CL#3@ 3.512];

[CL#4@ 0.362];

%cg#1.cw#4%

[CL#1@ -11.599];

[CL#2@ -1.663];

[CL#3@ -1.22];

[CL#4@ 2.04];

%cg#1.cw#5%

[CL#1@ -3.783];

[CL#2@ -4.263];

[CL#3@ -3.806];

[CL#4@ -3.587];

*! Group 2 (England)*

%cg#2.cw#1%

[CL#1@ 3.978];

[CL#2@ 0.292];

[CL#3@ -0.225];

[CL#4@ -9.78];

%cg#2.cw#2%

[CL#1@ -0.349];

[CL#2@ 2.546];

[CL#3@ 0.241];

[CL#4@ -0.475];

%cg#2.cw#3%

[CL#1@ -0.922];

[CL#2@ 0.948];

[CL#3@ 3.632];

[CL#4@ 0.666];

%cg#2.cw#4%

[CL#1@ -11.307];

[CL#2@ -1.176];

[CL#3@ -0.5];

[CL#4@ 2.339];

%cg#2.cw#5%

[CL#1@ -3.398];

[CL#2@ -3.577];

[CL#3@ -4.275];

[CL#4@ -3.691];

OUTPUT: STDYX CINTERVAL SVALUES RESIDUAL TECH1 TECH7;

**______________________________________________________________________________**

**Additional Instructions:**

Final proportions of L1 profiles that make up the L2 profiles are taken from the output section called: FINAL CLASS COUNTS AND PROPORTIONS FOR THE LATENT CLASS PATTERNS BASED ON ESTIMATED POSTERIOR PROBABILITIES. For example, the class count printed in the output for L1-profile 1 in L2-profile 1 for Australia (i.e., 1 1 1; value = 268.14) should be divided by the total count for all participants in L2-profile 1 for Australia (i.e., all profiles where CG=1 and CB=1; value = 3854.30) to reveal a percentage of the school-level profile (i.e., 6.96%. The same should be repeated for all profiles and all countries.

Then, assuming at least structural similarity was attained (in our case, distributional was attained and that output should be used), an overall percentage of the proportion of L1-profile 1 across both countries can be calculated by averaging the percentages estimated in Australia and England. For example, for 1 1 1 the percentage of L1-profile 1 in L2-profile 1 was 6.96% for Australia and 8.21% for England. Averaged, this results in 7.95% (rounded to 8% in our Results).

FINAL CLASS COUNTS AND PROPORTIONS FOR THE LATENT CLASS PATTERNS

BASED ON ESTIMATED POSTERIOR PROBABILITIES

Latent Class

Pattern

1 1 1 268.13931 0.03043

1 1 2 456.82554 0.05185

1 1 3 1783.62371 0.20243

1 1 4 636.53552 0.07224

1 1 5 709.17388 0.08049

1 2 1 464.10971 0.05267

1 2 2 582.25281 0.06608

1 2 3 568.73014 0.06455

1 2 4 331.68874 0.03764

1 2 5 609.96675 0.06923

2 1 1 102.72397 0.01166

2 1 2 163.64938 0.01857

2 1 3 568.55227 0.06453

2 1 4 193.87104 0.02200

2 1 5 221.74141 0.02517

2 2 1 218.07452 0.02475

2 2 2 288.95952 0.03280

2 2 3 245.51627 0.02786

2 2 4 133.35075 0.01513

2 2 5 263.51477 0.02991

**Mplus Input Syntax for L2 Profile Similarity Tests Step 4a: L2 Explanatory Similarity with Outcomes Freely Estimated Across Countries**

DATA: FILE IS "GLOBALDAT.csv";

VARIABLE: NAMES ARE PLBARRS SMISBEH TCOLLAB AUTONOMY TEFF TT2G01 TT2G05B

JOBSAT OCCCOMM SCHWGT IDCNTRY CPROB1 CPROB2 CPROB3 CPROB4 CPROB5

CL TCHWGT SCHLID;

USEVARIABLES = CL JSB OCCB;

NOMINAL = CL;

KNOWNCLASS = cg (IDCNTRY = 36 IDCNTRY = 926);

CLASSES = CG (2) CB (2) CW (5);

WITHIN = CL;

BETWEEN = CG CB JSB OCCB;

CLUSTER = SCHLID;

MISSING ARE *;

weight=TCHWGT;

wtscale = cluster;

bweight=SCHWGT;

bwtscale = sample;

DEFINE:

JSB=cluster_mean(JOBSAT);

OCCB=cluster_mean(OCCCOMM);

ANALYSIS:

TYPE = MIXTURE TWOLEVEL;

STARTS = 0;

MODEL:

%WITHIN%

%OVERALL%

%BETWEEN%

%OVERALL%

CW ON CB;

MODEL CG.CW:

%WITHIN%

*!Group 1 (Australia)*

%cg#1.cw#1%

[CL#1@ 4];

[CL#2@ -1.196];

[CL#3@ 0.12];

[CL#4@ -9.772];

%cg#1.cw#2%

[CL#1@ -1.079];

[CL#2@ 2.401];

[CL#3@ 0.492];

[CL#4@ -0.277];

%cg#1.cw#3%

[CL#1@ -1.152];

[CL#2@ 0.529];

[CL#3@ 3.512];

[CL#4@ 0.362];

%cg#1.cw#4%

[CL#1@ -11.599];

[CL#2@ -1.663];

[CL#3@ -1.22];

[CL#4@ 2.04];

%cg#1.cw#5%

[CL#1@ -3.783];

[CL#2@ -4.263];

[CL#3@ -3.806];

[CL#4@ -3.587];

*! Group 2 (England)*

%cg#2.cw#1%

[CL#1@ 3.978];

[CL#2@ 0.292];

[CL#3@ -0.225];

[CL#4@ -9.78];

%cg#2.cw#2%

[CL#1@ -0.349];

[CL#2@ 2.546];

[CL#3@ 0.241];

[CL#4@ -0.475];

%cg#2.cw#3%

[CL#1@ -0.922];

[CL#2@ 0.948];

[CL#3@ 3.632];

[CL#4@ 0.666];

%cg#2.cw#4%

[CL#1@ -11.307];

[CL#2@ -1.176];

[CL#3@ -0.5];

[CL#4@ 2.339];

%cg#2.cw#5%

[CL#1@ -3.398];

[CL#2@ -3.577];

[CL#3@ -4.275];

[CL#4@ -3.691];

*!Below is where differences in the outcomes at the school-level are tested across groups (i.e., the two countries)*

MODEL CG.CB:

%BETWEEN%

*! Group 1 (Australia)*

%cg#1.cb#1%

[JSB] (js1);

[OCCB] (oc1);

%cg#1.cb#2%

[JSB] (js2);

[OCCB] (oc2);

*! Group 2 (England)*

%cg#2.cb#1%

[JSB] (js3);

[OCCB] (oc3);

%cg#2.cb#2%

[JSB] (js4);

[OCCB] (oc4);

*!Model constraints allow for tests of mean differences*

MODEL CONSTRAINT:

NEW (job1v2 occ1v2 job3v4 occ3v4 job1v3 occ1v3 job2v4 occ2v4);

*!Comparisons across profiles in Australia*

job1v2 = js1-js2;

occ1v2 = oc1-oc2;

*!Comparisons across profiles in England*

job3v4 = js3-js4;

occ3v4 = oc3-oc4;

*!Comparisons across profiles across country (comparing like L2 profiles across Australia and England)*

job1v3 = js1-js3;

occ1v3 = oc1-oc3;

job2v4 = js2-js4;

occ2v4 = oc2-oc4;

**Mplus Input Syntax for L2 Profile Similarity Tests Step 4b: L2 Explanatory Similarity with Outcomes Constrained to Equality Across Countries**

DATA: FILE IS "GLOBALDAT.csv";

VARIABLE: NAMES ARE PLBARRS SMISBEH TCOLLAB AUTONOMY TEFF TT2G01 TT2G05B

JOBSAT OCCCOMM SCHWGT IDCNTRY CPROB1 CPROB2 CPROB3 CPROB4 CPROB5

CL TCHWGT SCHLID;

USEVARIABLES = CL JSB OCCB;

NOMINAL = CL;

KNOWNCLASS = cg (IDCNTRY = 36 IDCNTRY = 926);

CLASSES = CG (2) CB (2) CW (5);

WITHIN = CL;

BETWEEN = CG CB JSB OCCB;

CLUSTER = SCHLID;

MISSING ARE *;

weight=TCHWGT;

wtscale = cluster;

bweight=SCHWGT;

bwtscale = sample;

DEFINE:

JSB=cluster_mean(JOBSAT);

OCCB=cluster_mean(OCCCOMM);

ANALYSIS:

TYPE = MIXTURE TWOLEVEL;

STARTS = 0;

MODEL:

%WITHIN%

%OVERALL%

%BETWEEN%

%OVERALL%

CW ON CB;

MODEL CG.CW:

%WITHIN%

*!Group 1 (Australia)*

%cg#1.cw#1%

[CL#1@ 4];

[CL#2@ -1.196];

[CL#3@ 0.12];

[CL#4@ -9.772];

%cg#1.cw#2%

[CL#1@ -1.079];

[CL#2@ 2.401];

[CL#3@ 0.492];

[CL#4@ -0.277];

%cg#1.cw#3%

[CL#1@ -1.152];

[CL#2@ 0.529];

[CL#3@ 3.512];

[CL#4@ 0.362];

%cg#1.cw#4%

[CL#1@ -11.599];

[CL#2@ -1.663];

[CL#3@ -1.22];

[CL#4@ 2.04];

%cg#1.cw#5%

[CL#1@ -3.783];

[CL#2@ -4.263];

[CL#3@ -3.806];

[CL#4@ -3.587];

*! Group 2 (England)*

%cg#2.cw#1%

[CL#1@ 3.978];

[CL#2@ 0.292];

[CL#3@ -0.225];

[CL#4@ -9.78];

%cg#2.cw#2%

[CL#1@ -0.349];

[CL#2@ 2.546];

[CL#3@ 0.241];

[CL#4@ -0.475];

%cg#2.cw#3%

[CL#1@ -0.922];

[CL#2@ 0.948];

[CL#3@ 3.632];

[CL#4@ 0.666];

%cg#2.cw#4%

[CL#1@ -11.307];

[CL#2@ -1.176];

[CL#3@ -0.5];

[CL#4@ 2.339];

%cg#2.cw#5%

[CL#1@ -3.398];

[CL#2@ -3.577];

[CL#3@ -4.275];

[CL#4@ -3.691];

*!Below, the means of the outcomes are constrained to equality across group (i.e., the two countries)*

MODEL CG.CB:

%BETWEEN%

*! Group 1 (Australia)*

%cg#1.cb#1%

[JSB] (js1);

[OCCB] (oc1);

%cg#1.cb#2%

[JSB] (js2);

[OCCB] (oc2);

*! Group 2 (England)*

%cg#2.cb#1%

[JSB] (js1);

[OCCB] (oc1);

%cg#2.cb#2%

[JSB] (js2);

[OCCB] (oc2);
